# A cognitive nose? Evaluating working memory benchmarks in the olfactory domain

**DOI:** 10.1093/chemse/bjaf008

**Published:** 2025-03-10

**Authors:** Theresa L White, Nira Cedres, Jonas K Olofsson

**Affiliations:** Department of Psychology, Le Moyne College, Syracuse, NY, United States; Faculty of Health Sciences, University Fernando Pessoa Canarias, Las Palmas de Gran Canaria, Spain; Department of Psychology, Stockholm University, Stockholm, Sweden; Department of Psychology, Stockholm University, Stockholm, Sweden

**Keywords:** olfaction, sense of smell, smell, short-term memory, perception

## Abstract

Working memory (WM) processes are assumed to operate on a wide variety of sensory materials, yet WM research rarely extends beyond sight and hearing. In this systematic review, we integrate research from studies that address WM in olfaction, the sense of smell, spanning the last 50 yr (*N* = 44). We assessed whether 21 proposed “benchmarks” for WM generalize to olfactory WM. Seven benchmarks generalized to olfaction, whereas 2 failed to generalize. Evidence was insufficient to address the remaining 12 benchmarks (4 had mixed support and 8 were yet unaddressed). We conclude that the available evidence indicates that the sense of smell has a short-term memory system that mostly resembles WM processes in “higher” senses, although there are exceptions related to how olfactory WM performance is associated with other functions. We argue that researchers studying WM should explicitly consider evidence outside of the audio-visual senses when establishing theoretical frameworks. Further, we point out avenues for future research that may help close the remaining gaps in knowledge on this neglected topic.

## 1. Introduction

Working memory (WM) is the mental capacity to temporarily hold and process cognitive representations for use in action or thought (Cowan 2017). In human psychology, WM is arguably the paradigmatic domain of “higher cognition” as it is responsible for distributing a limited amount of processing resources among lower-level systems (see e.g. Hambrick et al. 2005, for review). Although there is no universal agreement on how exactly WM is organized (e.g. Morey 2018; Oberauer et al. 2018a; Cowan 2019; Norris 2019), a recent initiative (Oberauer et al. 2018a) aimed instead at building a consensus regarding empirical “benchmarks” that reflect findings that every theory in the field should be able to explain and that also should generalize across stimulus materials. The purpose of the current review is to evaluate whether these widely accepted benchmarks of WM extend to the sense of smell, olfaction. We will assess the extent to which the benchmarks fulfill the assumption of generalizability by using olfaction as a criterion. Our evaluation will also address the question of whether human olfaction has a WM capacity that operates in a similar way as “higher” visual and auditory senses.

Olfaction provides a way to evaluate whether or not WM processes are similar for stimuli presented in different sensory channels (e.g. visual, verbal, olfactory). The neuroanatomical pathways of olfaction, located within the archicortex, deviate from the neocortical pathways that dominate audition and vision, which potentially affects the interaction of olfaction with higher cognition. Theories of WM are typically based on research with verbal or visuospatial stimuli, but WM is regarded as indicative of a domain-general cognitive ability. For example, WM is highly correlated with general intelligence, and it is believed that WM reflects the general processing capacity needed for cognitive problem-solving (Kane et al. 2005). Influential models of WM, such as the modal model (Baddeley and Hitch 1974) include sensory input modules that interact with a domain-general “central executive.” Assumptions of domain-general processing in WM are sometimes implicit; as Shah and Miyake (1999) noted, although theoretical frameworks may be developed to account only for results using a specific set of stimuli, this limitation is not always clearly stated, leading other researchers to mistakenly assume that the framework has a domain-general scope. Under the (implicit or explicit) assumptions of domain-general WM processes, similar results should be found when WM tasks are adapted to various sensory stimuli. Memory processes quite clearly occur in sensory systems other than vision and hearing. Yet, researchers studying WM have primarily considered verbal or visuospatial stimuli, rather than examining memory for other sensory systems, such as flavors, textures, or odors (e.g. Engen et al. 1973; Johnson and Miles 2009; Morimoto 2020).

The sense of olfaction is rarely considered in overviews of WM (e.g. D’Esposito and Postle 2015; Norris 2017; Cowan 2019; but see Baddeley 2012 for a brief note). Historically, leading thinkers often dismissed the cognitive capabilities of olfaction (see e.g. Barwich 2020, for review). For example, Plato argued that “the varieties of smell (...) are only distinguished as painful or pleasant,” whereas Immanuel Kant stated that among the senses, olfaction was “the most ungrateful and also seems the most dispensable” (Plato, tran. 2009, p. 147; Kant 1798/2006). The French philosopher Étienne Bonnot de Condillac wrote that smell “appears to contribute least to the cognitions of the human mind” (Condillac 1754/1930). The pioneering 19th-century neurologist Paul Broca famously classified some animals as “osmatic” (olfactory-oriented), whereas humans were considered “anosmatic” (not olfactory-oriented), which later morphed into the view that humans are poor smellers (Broca 1879; as cited by McGann 2017). Interestingly, Broca even suggested an evolutionary trade-off such that development of the “highest intellectual functions” was linked to a reduction of the olfactory system. Broca wrote that in primate evolution, “intelligence (...) gained supremacy over this bestial sense (...) by two concurrent anatomical events: the advanced development of the frontal lobe, and the atrophy of the olfactory lobe” (Broca 1878; as cited by Pessoa and Hof 2015). This perceived trade-off between olfaction and higher cognition might have discouraged researchers from investigating their interactions.

Besides historical prejudice, or perhaps difficulties in managing the presentation of the stimulus, there is no a priori reason for leaving olfaction out of the study of higher cognitive abilities. The human sense of smell is highly sensitive (Laska 2017; McGann 2017) and, like other sensory systems, provides important information daily. As one of the major senses, olfaction is of critical importance for food intake (Yeomans 2000; Stevenson 2010), the avoidance of hazardous chemicals (Stevens et al. 1987), well-being (Croy et al. 2014), and emotional attachment (VanHatten et al. 2018). Olfaction’s importance in these domains was underscored during the COVID-19 pandemic where olfactory loss was a common symptom and difficulties with food, safety, and intimacy arose for many people (Parma et al. 2020). Similar to vision and hearing, the human olfactory system is capable of cognitive abilities, such as recognizing the stimulus source in a multiple-choice setting (Doty et al. 1984), recognizing an odor from a background of other smells (Li et al. 2006), making rapid perceptual classifications (Olofsson et al. 2012), and guiding spatial navigation (Porter et al. 2007). Methods for exact, computerized, olfactory stimulation, and assessment are increasingly available, and these devices enable careful, time-sensitive cognitive experimentation with odors (Lundström et al. 2010; Niedenthal et al. 2021). Given these abilities and advances in olfactory presentation techniques, olfaction should be considered alongside the cognition of other sensory systems. Comprehensive theories of WM should include all of the major senses both for completeness and in order to assess the generalizability of proposed higher cognitive functions. Here, we will provide a comprehensive overview of olfactory working memory (OWM) research in order to address whether WM benchmarks generalize to the olfactory domain. This question, we argue, provides a critical test of the domain-general assumption in WM research, and at the same time, a reconsideration of the old notion that higher cognitive capabilities are sparse in the olfactory system, in light of aggregated empirical evidence. In addition, our comprehensive OWM review may facilitate theory development in the field of olfactory-based cognition. There are several unresolved theoretical issues in the field of olfactory cognition. It is debated whether OWM of brief durations should be characterized as a passive buffer system (e.g. Zelano et al. 2009; Wenzel et al. 2021) or an active OWM system (e.g. White 2009; Moss et al. 2019). There is also no consensus regarding to what extent humans are able to imagine, mentally elicit, the qualities of well-known odors (see Arshamian and Larsson 2014, for review). As OWM processes would likely also be involved in many other tasks, such as imagery, a systematic evaluation of the available evidence for OWM might facilitate theoretical development in the field of human olfaction more generally.

### 1.1. Benchmarks of WM

Our examination of a putative OWM is organized according to the comprehensive list of “benchmarks” that was provided by Oberauer and colleagues (2018a). These benchmarks are sets of experimental findings that are considered theoretically informative, empirically robust, and that are purported to generalize across stimulus materials and methodologies. Oberauer and colleagues (2018a) generated the initial set of benchmarks through a process that included 2 workshops with experts and a survey of researchers on the topic. Although not universally agreed upon (Logie 2018; Oberauer et al. 2018b; Vandierendonck 2018), the benchmarks achieved consensus among the many involved researchers and were intended to provide an empirical “common ground” for theoretical discussions in the field of WM. The ambition was also that the benchmarks would be subject to revision as more data became available. Oberauer and colleagues (2018a) evaluated and classified each benchmark, with those designated as “A” considered well-established “across paradigms and content domains,” “B” being intermediate, and “C” being the least well-established. Although their approach did not explicitly motivate the exclusion of any particular stimulus type, the benchmarks were solely based on findings from verbal and visuospatial materials. Oberauer and colleagues (2018a, p. 917) were aware of this limitation and suggested that validating these benchmarks with stimuli from other sensory modalities could increase the generality of the findings. Thus, although findings with olfactory stimuli were not considered in designating these WM benchmarks, an examination of the OWM literature would be valuable in informing WM theory.

In the present paper, we focus on the 21 “A-level” WM benchmarks (see Table 1), which are proposed to generalize across content domains, and ask: How do results from olfaction meet these benchmarks (Oberauer et al. 2018a)? Following a well-defined systematic assessment of the literature, we will briefly describe each benchmark (maintaining the title assigned by Oberauer et al. 2018a), and then we will evaluate the olfactory evidence based on (i) whether or not there is relevant empirical support and (ii) whether or not the data supports the benchmark. In a concluding section, we summarize and discuss the implications of our findings and point out opportunities for future research.

**Table 1.** “A” level WM Benchmarks (Oberauer et al. 2018a) and associated numbering in the present paper.

Benchmark number	Benchmark
1	Set-Size Effects on Accuracy
2	Set-Size Effects on Retrieval Latency
3	The Effects of Filled Retention Intervals
4	Primacy and Recency Effects on Accuracy
5	Confusions of Target Items with Other Items in a Memory Set
6	Locality Constraint on Transpositions
7	Effects Within and Across Domains in a Multiple Memory-Set Effect
8	Disruption of Memory by Processing in the Same Domain
9	Disruption of Memory by Processing in Another Domain
10	Effect of Cognitive Load of the Processing Demand
11	Phonological Similarity
12	Effects of Distinctiveness and of Grouping: Grouped Lists are Better Recalled
13	Prioritization of Information in WM: Item-Switch Effects
14	Effects of Chunking
15	Hebb Repetition Effect
16	Positive Manifold
17	Correlation Between WM and Attention Indicators
18	Correlation of WM with Fluid Intelligence
19	Dissociable Neural Substrates of Different Content Domains
20	Preserved WM in Amnesia
21	Measures of Neural Activity Track Amount of Information in WM

## 2. Materials and methods

### 2.1. Definition of OWM

We defined OWM as a specific system or set of processes that hold mental representations of a sequence of experimentally presented odor stimuli; these representations should be temporarily available for use in thought or action over brief time intervals (maximum of 3 min). As a general rule, any task that would be called a WM task if it was performed with audio/visual materials, would also be considered an OWM task if performed with odors. We decided to have a restrictive definition of OWM, such that only tasks designed to test multiple odors were included. We recognize that WM may affect performance on a wide variety of odor tasks, such as identification, odor quality discrimination, and detection, or cross-modal tasks, but such tasks were not included.

### 2.2 Search strategy

The present study was performed according to the Preferred Reporting Items for Systematic reviews and Meta-Analyses (PRISMA) statement, which provides detailed guidelines for reporting results in systematic reviews and meta-analyses in health sciences (Moher et al. 2009). We searched the electronic databases of PubMed, Web of Sciences (WoS), and PsycINFO in December 2023. The search strategy was built for each database using a combination of free terms representing the central concepts of the study “working memory” and “olfaction” to be present in the title and/or the abstract without additional limits for publication date.

### 2.3. Studies selection

**Fig. 1.**
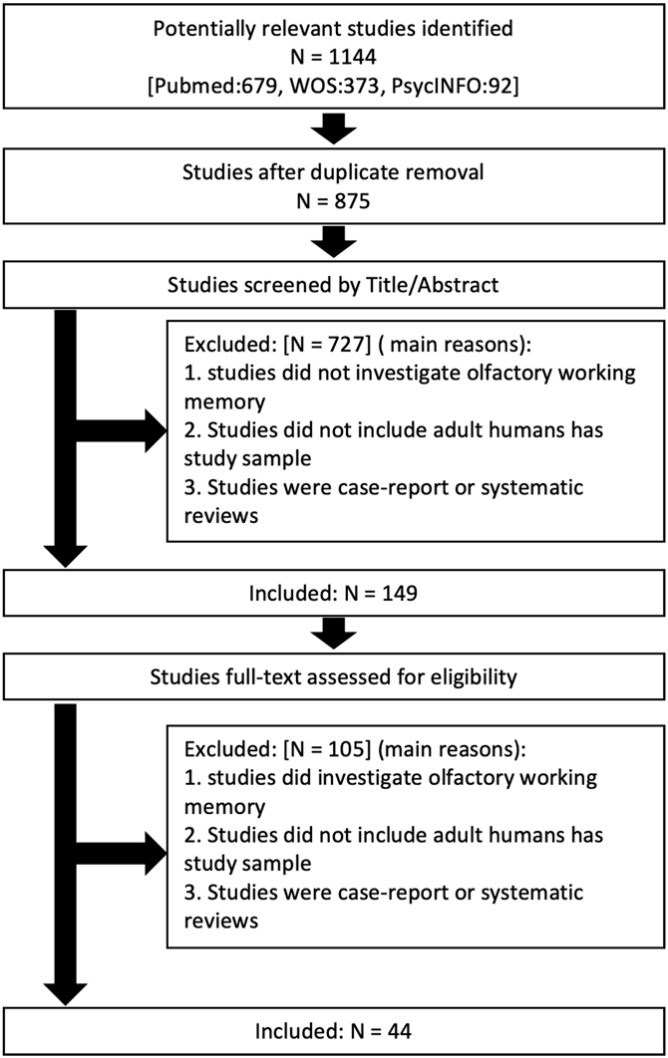
Flowchart of search and article screening procedure.

The current review includes peer-reviewed original research articles that (i) cross-sectionally evaluate the performance in OWM (as defined above), (ii) include adult human participant samples, and (iii) are published in English, which enabled the authors to have unmediated access to the content of the studies and thus achieve valid data extraction and quality control (e.g. inter-rater consensus on key decisions). We excluded (i) cases-studies, reviews, animal model studies, longitudinal studies, and poster or conference presentations; (ii) studies that did not investigate OWM; (iii) included children, patients with nasal obstruction and/or structural anosmia; (iv) and studies that were not published in English (Fig. 1). Study selection was performed by the 3 authors, dividing the number of studies equally, after an initial training where 51 studies were collectively screened for inclusion/exclusion. All disagreements were resolved through discussion, and the eligibility and inclusion of the final set of studies was achieved via consensus among all 3 authors.

A total of 1144 studies were identified in the initial search and 31 studies were identified through other sources. After removing duplicates between databases (*n* = 269) and applying the inclusion/exclusion criteria for title and abstract, a total of 149 studies were assessed full-text for eligibility. Finally, a total of 44 studies were selected for their inclusion in the current systematic review.

### 2.4. Data extraction

Data extraction was performed by all 3 of the authors. A data extraction sheet was created covering the following aspects: authors’ names and publication year, brief description of the OWM task, WM benchmark associated. To assess the methodological quality of the studies included, we used the JBI critical appraisal tool (https://jbi.global/critical-appraisal-tools), which allows for a qualitative estimation and standardized analysis of both the quality and reliability of different types of studies, following a series of criteria divided into several areas that facilitate the systematic extraction of data of interest. We evaluated each article on 8 different criteria concerning methodology (see *Critical Appraisal of Reviewed Studies*, Supplementary Table S1, https://osf.io/kg6fy/?view_only=7eb94c594e1e4836b867815b5e61464f). The criteria were: (i) Were the criteria for inclusion in the sample clearly defined? (ii) Were the study subjects and the setting described in detail? (iii) Was the exposure measured in a valid and reliable way? (iv) Were objective, standard criteria used for measurement of the condition? (v) Were confounding factors identified? (vi) Were strategies to deal with confounding factors stated? (vii) Were the outcomes measured in a valid and reliable way? (viii) Was appropriate statistical analysis used? All 44 of the articles met at least 7 out of 8 criteria, and all articles were retained.

In keeping with the main focus of the present paper, we evaluated each study regarding whether or not they (i) provided results that were relevant for any of the benchmarks and (ii) supported the generalization of these benchmarks to the olfactory domain. Fulfillment of these criteria were decided upon by the authors through consensus. Given that the available experimental literature may be ambiguous in demonstrating a particular benchmark outcome, the evidence was classified in 4 ways: support, unsupported, mixed support, or unaddressed. Olfactory experimental evidence was classified as *supporting* a given benchmark if the preponderance of the relevant evidence (a proportion of at least 2/3) had results consistent with those outlined by Oberauer et al. (2018a). A classification of *unsupported* was given if the preponderance of the olfactory evidence didn’t support a benchmark. Results were classified as offering *mixed support* if results fell in-between “supported” and “unsupported.” Benchmarks were classified as *unaddressed* if none of our listed publications addressed them. For benchmarks that involved more than one empirical phenomenon (e.g. the *serial position* benchmark involves both primacy and recency effects), all of the phenomena should meet the criteria of above (2/3 of the evidence) for either a “supported” or “unsupported” classification to be made, otherwise the results were deemed to be “mixed.” The 2/3 criterion was used to categorize the evidential trend across studies within each benchmark. We acknowledge there is no objective way to establish such a criterion, but since our systematic review approach enables full methodological transparency, readers are free to reanalyze our data and reinterpret results with their own preferred criteria. For each study, only results reaching traditional levels of statistical significance were regarded as providing evidence for an effect, in accordance with Oberauer and colleagues (2018a).

### 2.5. Transparency and openness

Our methodology and evaluation criteria are described in the manuscript text above and in our Supplementary Table S1, https://osf.io/kg6fy/?view_only=7eb94c594e1e4836b867815b5e61464f. Below, we reference all selected studies (see Table 2) such that the original results can be easily accessed. The study was not preregistered.

**Table 2.** OWM tasks and benchmark associated with the included studies organized by year of publication.

Authors (Year of publication)	Task	Benchmarks
Engen et al. (1973)	Short-term odor memory with varying filled and unfilled delay time and set size	1, 3
Jones et al. (1975)	Odor 1-back with immediate or 30-s delay	3
Jones et al. (1978)	Short-term memory for odor sets that vary in size	1
Mair et al. (1980)	Recognition memory tested after varying filled time delays	3
Eskenazi et al. (1983)	Odor recognition memory with varying delays	20
Walk and Johns (1984)	Odor matching to a recent sample of 2 odors after interference from distractors of both the same and different types of stimuli	8, 9
Murphy et al. (1991)	Both experiments 3 and experiment 4 entail a delayed match to sample task.	3, 9
Doty et al. (1994)	Odor recognition after varying filled intervals, including matching a target odor to a set of 4 odors	None
Jehl et al. (1994)	Odor 1-back with varying delay and similarity	None
Annett et al. (1995)	Odor memory recall and recognition with concurrent verbal and visual suppression tasks during encoding	9
Annett and Lorimer (1995)	Odor recognition memory with serial position analysis	4
Bromley and Doty (1995)	Uninasally and binasally presented odor sequences for matching after a variety of delay intervals that were filled with backward counting	3
Doty et al. (1995)	Odor recognition including matching a target odor to a set of 4 odors	None
White and Treisman (1997)	Odor item and order recognition in 5-odor sequences that show serial position effects	4, 5
Dade et al. (1998)	Short-term odor recognition memory (no exact time estimates provided)	19
White et al. (1998)	Recall of odors that varied in perceptual and phonological similarity presented in sequences of 5	11
Miles and Jenkins (2000)	Odor recall with serial position effects	3, 4
Reed (2000)	Odor recognition with serial position effects	4
Dade et al. (2001)	Odor 2-back	19
Danthiir et al. (2001)	Odor memory involving a detection of change across 2 odor sequences	16, 18
Dade et al. (2002)	Odor recognition memory with 3-min delay	19
Choudhury et al. (2003)	Odor match to sample following various delay intervals that were filled with backward counting	3
Levy et al. (2003)	Odor span, iterative match to sample	20
Zucco (2003)	Experiment 2 is an odor recognition task, with an interference task that followed list presentation	8, 9
Dacremont and Valentin (2004)	Short-term odor memory span	1
Miles and Hodder (2005)	Serial position effects in recognition memory for odors, some with articulatory suppression	4, 9
Andrade and Donaldson (2007)	Odor match to sample during verbal distraction; Concurrent tasks for verbal, visual, and odor memory	7, 8, 9
Johnson and Miles (2007)	Pairs of odors presented for recognition as to the correct order to examine serial position	4
Doty et al. (2008)	Odor recognition with varying filled intervals, including matching a target odor to a set of 4 odors	3
Yeshurun et al. (2008)	Odor-odor matching with 12-s delay	None
Johnson and Miles (2009)	Serial position effects for odors and other stimuli	4
Zelano et al. (2009)	Delayed match to sample with odors that are either easily named or not	19
Jönsson et al. (2011)	Odor 2-back	None
Valentin et al. (2011)	Short-term odor recognition memory test for varying set-sizes after a 20-s unfilled delay	1
Zucco et al. (2011)	Delayed match to sample with odors	None
Johnson et al. (2013)	Serial order reconstruction task in odors	4, 15
Lenk et al. (2014)	Odor intensity comparison including EEG and varying distraction	19
Doty et al. (2015)	Delayed match to sample with odors	3, 16
MacQueen and Drobes (2017)	Odor span, involving an iterative match to sample	1, 16
Moss et al. (2018)	Matching an odor probe that varied in serial position at presentation after a 4-odor sequence	4
Moss et al. (2019)	Odor 2-back, some with concurrent articulation	9
Wenzel et al. (2021)	Matching target odors to recently encoded odors after various filled delay intervals	3
Yang et al. (2021)	Sternberg WM task with 3 odors	4, 5
Johnson and Allen (2022)	Serial position effects in odor-color association memory	4, 13

### 2.6. Ethics statement

Our study was carried out in accordance with the Declaration of Helsinki for Medical Research Involving Human Subjects. All data in this systematic review are research articles in the public domain, and our work is thus exempt from ethical review.

## 3. Results and discussion

A total of 44 research articles focused on the study of OWM. These studies were published between 1973 and 2023 and were included in the current systematic review (Table 2). Since there is some evidence that the nameablity of the olfactory stimuli in the WM study may influence experimental outcomes (e.g. Moss et al. 2018; Wenzel et al. 2021), we have noted the stimulus descriptions in each study, although nameability was rarely assessed in the original studies (see Supplementary Table S2). Our supplemental materials also include a version of Table 2 which is organized according to benchmarks, rather than research studies in chronological order (Supplementary Table 3). Below, the 21 WM benchmarks are defined and assessed based on the evidence from the OWM literature. Please note that the numbers delineating the benchmarks in the present paper do not directly correspond to the numbering system provided by Oberauer et al. (2018a). Thirty-eight studies address at least one benchmark, but there are 6 that does not address any of the 21 benchmarks. In the following, each benchmark is defined along with a discussion of the results of research in the olfactory domain.

### 3.1. Set-size effects on accuracy

Accuracy in performing WM tasks is reduced as the number of items to remember increases (e.g. Murdock 1968). This finding has been reported across a broad range of visual and auditory stimuli (e.g. Crannell and Parrish 1957; Shah and Miyake 1996; Luck and Vogel 1997) and experimental paradigms (e.g. Murdock 1968; Wilken and Ma 2004; Cortis et al. 2015; Woods et al. 2016); however, the effect can be mitigated in a recognition task where each stimulus is only used as a probe or test stimulus only once, which minimizes the effects of proactive interference (Endress and Potter 2014).

**Fig. 2.**
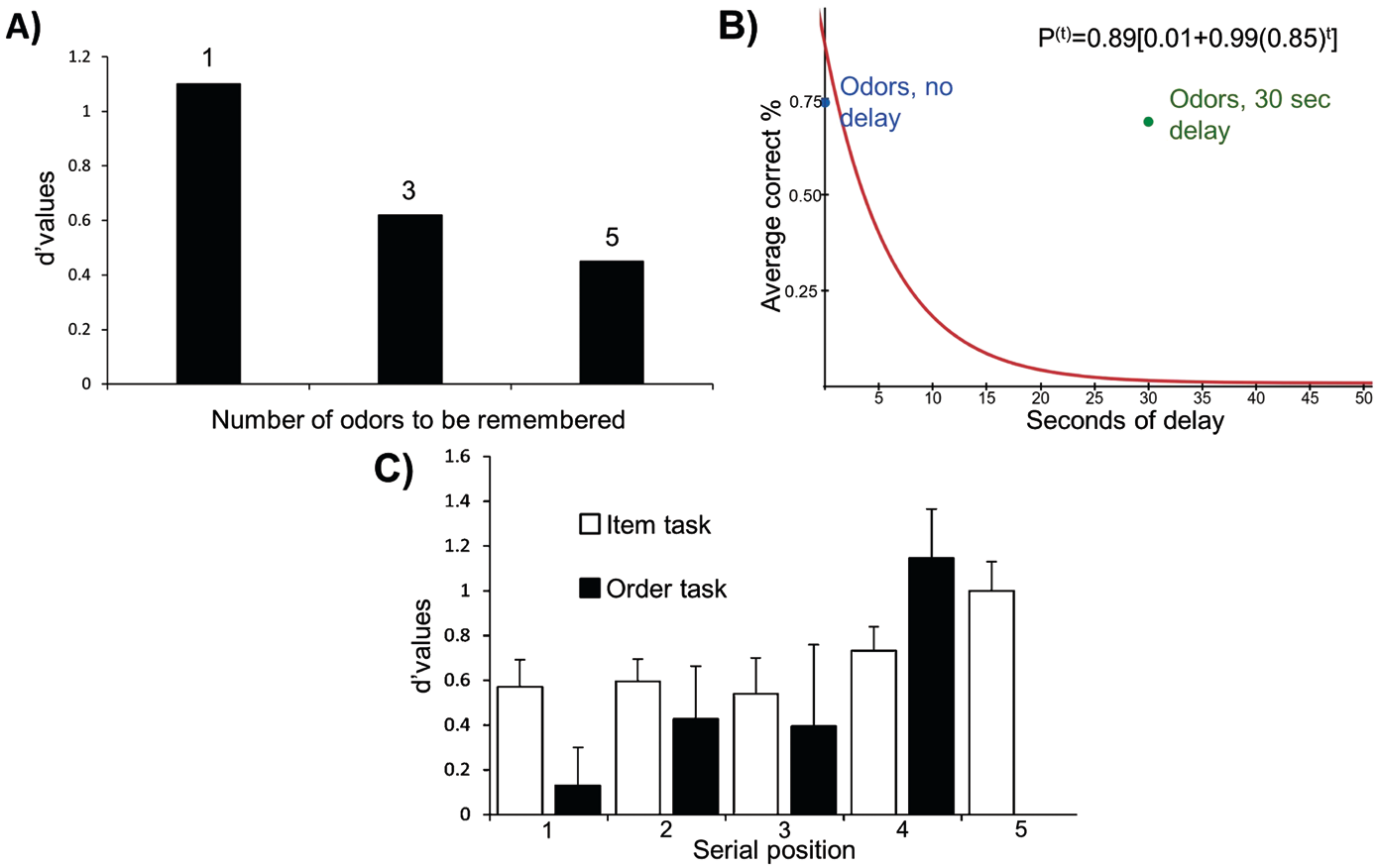
Exemplar results that illustrate benchmarks 1, 3, 4, and 5. *Note*. a) Set-size effects on accuracy (adapted from Jones et al. 1978). b) Verbal WM performance, as reported by Peterson and Peterson (1959), with additional data points reflecting OWM performance in Wenzel et al. (2021). c) Results reported in White and Treisman (1997), experiment 3 (mean d’ scores + standard errors). Note that only the order task shows a significant effect of recency, and that the item task shows generally better performance than the order task.

Five studies demonstrated this phenomenon with olfactory stimuli (Engen et al. 1973; Jones et al. 1978; Dacremont and Valentin 2004; MacQueen and Drobes 2017; Valentin et al. 2011; see Table 2). In perhaps the earliest investigation, Engen and colleagues (1973) examined recognition memory for briefly presented olfactory stimuli (adapted from the Brown-Peterson task—Brown 1958; Peterson and Peterson 1959). Subjects were presented with either 1 or 5 odors to be remembered. After a brief delay interval, subjects were asked to recognize whether a probe was a member of the original list or a distractor. Recognition was better for the 1-odor condition than the 5-odor condition, which is in keeping with findings from verbal and visual memory (Murdock 1961). This result was later replicated and extended, such that one item is remembered better than either 3 or 5 items, and that 3 items are remembered better than 5 (Jones et al. 1978; see Fig. 2a) or 12 items (Valentin et al. 2011). Memory span tasks (Dacremont and Valentin 2004; MacQueen and Drobes 2017) demonstrated negative relationships between accuracy and the number of odorants to be remembered, further illustrating the influence of set size. No study included contradictory results.

Similar to verbal and visual stimuli, olfactory stimuli seem to be restricted by a limited capacity mechanism, and the benchmark is thus *supported*.

### 3.2. Set-size effects on retrieval latency

Set-size determines the speed at which an item in WM can be retrieved (Sternberg 1966). Behavioral responses drawing on information in WM become slower as there are more items in a list to remember (e.g. Oberauer and Kliegl 2001).

Our review of the olfactory literature showed that unfortunately, the time to retrieve items is seldom recorded in OWM tasks. This lack of information is likely because odors are often presented in bottles held under the nose of the participant by an experimenter; this simple setup is not suitable for time-sensitive response analysis. There is no olfactory research that speaks to this benchmark and it remains *unaddressed* (see Table 2). As olfactometers and computer interfaces are becoming more widely used in olfactory experiments, more precise timing may allow future research to address this benchmark.

### 3.3. The effects of filled retention intervals

An increasing retention interval length between stimulus presentation and retrieval decreases memory performance when participants must perform an interfering task (e.g. counting backwards) during that interval (Peterson and Peterson 1959). This effect arises because events that occur between the stimulus list and the memory test can be distracting and prevent rehearsal (Brown 1958); the effect is greater in longer intervals (e.g. Lewandowsky et al. 2010). Interestingly, the duration of a retention interval filled with verbal stimuli also interferes with recognition memory for visual-spatial stimuli, such as unconventional symbols (Ricker and Cowan 2010) or corsi-block performance (Kopelman and Stanhope 1997).

Ten OWM studies were relevant to this benchmark; they each varied filled retention intervals systematically (see Table 2). In contrast to verbal or visual information, WM for olfactory stimuli does not seem to be affected by extending an interval where the participant is occupied by verbal counting (Engen et al. 1973; Jones et al. 1975; Mair et al. 1980; Choudhury et al. 2003; Doty et al. 2008, 2015; Wenzel et al. 2021). The finding that the length of an interval filled with verbal material does not affect OWM is quite robust, though a few studies have shown an effect of verbal interference. One study comparing memory for ameboid shapes to that of odorants found a main effect of backward counting in the retention interval (Murphy et al. 1991); another article demonstrated a memory decline effect related to the length of the filled retention interval when participants were tested uninasally as opposed to binasally (Bromley and Doty 1995). Figure 2b shows the results reported for verbal memory by Peterson and Peterson (1959), along with OWM performance from one study (Wenzel et al. 2021) plotted on the same graph; this figure illustrates that recognition of probe odors seems unaffected by the duration of the counting-filled retention interval up to 30 s.

OWM deviates from results obtained in verbal memory with this benchmark, and there are several different possibilities as to the underlying reason for such a difference. Odors are processed more slowly than visual or auditory stimuli (Laing and MacLeod 1992), so one possibility is that more time is necessary for distraction to occur; the 30 s or so typically tested may not be sufficient. In support of this idea, a study that did not meet our inclusion criteria found that a verbal distractor task lasting 2 min reduced odor memory, whereas one lasting only 30 s had no effect (Gilmore 1991). Olfactory memory is regarded as notoriously long-lived, with some studies reporting minimal decrement over a period of months or more (e.g. Engen and Ross 1973; Lawless 1978; but see Olsson et al. 2009), so this stability may have affected retention of stimuli in OWM. Another possibility is that the type of interference chosen in previous studies may not prevent the rehearsal of olfactory stimuli. The nature of the interference may well matter, as evidence regarding interference based on type of stimuli in OWM tasks is decidedly mixed. Although the length of delay interval seems to be unaffected by counting, it is possible that other types of stimuli, such as visual (Moss et al. 2019; Exp. 2; Annett and Leslie 1996) or olfactory (Walk and Johns 1984; Miles and Jenkins 2000; Andrade and Donaldson 2007) stimuli might be more effective as a distractor task. A final possible reason that increasing the time of a filled delay does not affect OWM may be that mental rehearsal is not possible for olfactory sensations, as suggested by several theorists (Engen et al. 1973; Zucco and Job 2012), due to the stability of stored olfactory representations. Future research may address which of these interpretations, if any, are correct. In sum, there is strong evidence that OWM yields different results from that of visual and verbal WM, although there is no agreement on the underlying cause of this difference. This benchmark is *unsupported* in olfaction.

### 3.4. Primacy and recency effects on accuracy

In a list of items to be remembered, items at the beginning (primacy effect) or the end (recency effect) are better recalled than those in the middle of the list (e.g. Lashley 1951; Donaldson and Glathe 1969). The recency effect can be reduced or eliminated through the inclusion of a distractor (suffix effect) at the end of the list (Crowder 1967). Although often tested with recall, serial position effects have also been observed in recognition tests for verbal material (e.g. Oberauer 2003a), particularly when the task involves attending to a particular list position in order to respond (e.g. Fuchs 1969). While full serial position curves have been frequently observed for verbal and spatial material, evidence for the serial order effect in the visual domain is not yet firmly established (see Hurlstone et al. 2014 for review). Memory for serial positions of visual or spatial stimuli has often been assessed by serial recall, where participants are given lists visually and are asked to respond by speaking, writing, or typing the names of the items (such as numbers or letters) that had been on the list in order.

Eleven OWM studies were relevant to this benchmark (see Table 2). In order to minimize the effects of verbalization (and the influence of verbal memory), the serial position effect has been investigated with recognition tasks involving olfactory stimuli, such as the item probe task (White and Treisman 1997; Johnson and Miles 2007), the order probe task (White and Treisman 1997; see Fig. 2c), serial reconstruction (Johnson et al. 2013), 2-alternative forced choice (Johnson and Miles 2007), cross-modal binding (Johnson and Allen 2022), and the n-back task (Moss et al. 2018). The evidence points to a serial position curve that is characterized by a recency effect without a primacy effect (see Fig. 2c) for olfactory stimuli (Annett and Lorimer 1995; White and Treisman 1997; Miles and Hodder 2005; Johnson and Miles 2007; Johnson and Miles 2009; Johnson et al. 2013; Yang et al. 2021; Johnson and Allen 2022; Experiment 3; Johnson and Allen 2022), though some experiments have failed to demonstrate either primacy or recency effects (Miles and Hodder 2005; Johnson and Miles 2007, 2009). To date, one multiexperiment paper has demonstrated a full serial position curve (both primacy and recency effects) using a recognition paradigm for olfactory stimuli (Reed 2000). Attempts to replicate these results were successful in one case (Miles and Hodder 2005, Experiment 3) but unsuccessful in 8 other experiments (Miles and Hodder 2005; Johnson and Miles 2007).

Although less relevant to the present benchmark, it is worth noting that when a serial recall technique is employed with olfactory stimuli, a full serial position curve can be observed that demonstrates both primacy and recency effects (Annett and Lorimer 1995; Miles and Jenkins 2000), as well as a reduction in recency due to the presentation of an olfactory suffix (Miles and Jenkins 2000). However, this technique forces participants to recode an olfactory stimulus verbally, and simply to recall the verbal code (Herz and Engen 1996; Zucco 2007), thus the result is likely affected by the verbal task demands.

One may ask why odor memory would favor recency. In contrast to other stimulus modalities, where serial order is crucial for the acquisition of skills, such as reading (Gathercole and Baddeley 1990) and social behaviors (Agam et al. 2005, 2007; Baddeley 2007), the main purpose of olfactory serial memory may simply be to detect stimulus novelty (Jehl et al. 1994; Köster et al. 2014). However, there are other potential explanations for the shape of the olfactory serial position curve.

The fact that an olfactory primacy effect is not reliably achieved in these recognition paradigms may well be due to the task demands of recognition as compared to recall, since immediate recognition tests of memory for other types of stimuli is often characterized by the pattern of recency without primacy (e.g. Monsell 1978; McElree and Dosher 1989). Another possible explanation for the absence of the primacy effect may be constraints on olfactory cognition; since the primacy portion of the curve is thought to arise from differential rehearsal (Waugh and Norman 1965) that leads to consolidation to LTM, it is possible that the consolidation process differs for olfactory stimuli and that rehearsal capacity might be especially sparse for odors (Engen et al. 1973; Gabassi and Zanuttini 1983). Still other alternative interpretations for the recency-only olfactory serial position pattern have been offered, such as differences in the psychological distinctiveness of stimuli (Hay et al. 2007; Johnson and Miles 2009) or the number of items between the presentation of a target and the test of the item (“lag”; Johnson and Miles 2007).

We conclude that there is *mixed* evidence for this benchmark, as serial memory for odors typically yields a recency effect, similar to that of other materials, but primacy effects are not typically observed. Future research will hopefully explore the reason for this particular pattern of serial memory in olfaction.

### 3.5. Confusions of target items with other items in a memory set

In serial WM tasks, the items to be remembered are often confused such that the order of the memorized items is transposed (e.g. Fuchs 1969; Henson et al. 1996); in other words, the correct list members are recalled, but they are incorrect in terms of their order. In a recognition task, this can be reflected in terms of both increased error rates and slower response latencies (e.g. Oberauer 2003a; Smyth et al. 2005; Donkin et al. 2015) to probes that were a part of the presented list but are in an incorrect order (order error) compared to probes that were not part of the initially presented list (item error).

Two OWM studies were relevant for this benchmark (White and Treisman 1997; Yang et al. 2021). Very few studies have reported both item and order WM errors for olfactory stimuli. White and Treisman (1997) examined 2 different types of odor memory probes that were presented after a set of 5 odors: One type of probe tested item memory by asking whether or not the probe had appeared in the presentation list, and the other type of probe tested order memory by presenting 2 adjacent odors and asking the participant whether the order was correct. On average, the d’ scores for the item tasks were higher than the d’ scores for the order task, implying that the order of odors are often confused, supporting this benchmark (see Fig. 2c). A more recent study (Yang et al. 2021) reported results that initially seemed to dispute this finding, as the accuracy levels were similar for both the item and the order task. Here, however, the 2 tasks were not independent in this study; only people who identified that an odor was a part of the original list (item information) were asked for the serial position of the odor in the list (order information). Thus, while 60% accuracy could identify that it was previously seen, only 60% of that 60% (or 36%) correctly identified the location of the odor in the list. This interpretation leads us to conclude the studies were in fact in agreement, and we regard this benchmark as *supported* by the limited olfactory research that has examined it.

### 3.6. Locality constraint on transpositions

In a serial memory task, the distractors nearest to the target are more likely to be confused with it than those from other serial positions (Henson 1996). So, errors are more likely to involve transpositions with list items in positions adjacent to the target position, relative to positions farther away from the target. The errors form a gradient, so that the more displaced an item is from the target, the less likely the 2 are to be confused with each other (e.g. Smyth et al. 2005). In n-back tasks, where participants are asked to determine whether each item (e.g. letter) in a list matches an item that was presented *n-* positions prior to it (e.g. in a 2-back version, matches are defined as 2 positions prior to the item). When the 2 items do not match, the participant is more likely to erroneously respond that they do (false alarm) to *n* − 1 and *n* + 1 lure probes, as opposed to items farther away (e.g. McElree 2001; Jonides and Nee 2006).

No studies from our review provide direct evidence for or against this phenomenon. However, at least 3 OWM studies have been conducted that could have produced data relevant for this benchmark if it were possible to reexamine the data (Dade et al 2001; Jönsson et al 2011; Moss et al 2019). All 3 studies investigated performance on an olfactory 2-back task. While the rate of false alarms was reported in these studies (Jönsson et al. 2011; Moss et al. 2019), it was not reported by location of the error from the target. Moss et al (2019, Exp 4) reported the false alarm rates for the *n* − 1 and *n* + 1 lures, but they did not report the other positions for comparison, as this was not the focus of the study. Thus, although it is possible that evidence regarding this benchmark may exist for OWM, we regard this benchmark as *unaddressed*.

### 3.7. Effects within and across domains in a multiple memory-set effect

One of the key aspects of the modal model of WM is the separate buffers for holding information that arises from specific stimulus domains (Baddeley and Hitch 1974). Support for these buffers comes from the ability of people to simultaneously perform memory tasks for verbal and visuospatial materials (Logie 2011; Baddeley 2012), though others would suggest that these dual-task deficits do not require specialized stores (Morey 2018). Although WM performance for 2 sets of items is worse than memory for one set (Cowan and Morey 2007), people have more accurate memory when the sets contain stimuli from different domains than when they are comprised of information from the same domain (Logie et al. 1990; Fougnie and Marois 2006; Cowan and Morey 2007).

**Fig. 3.**
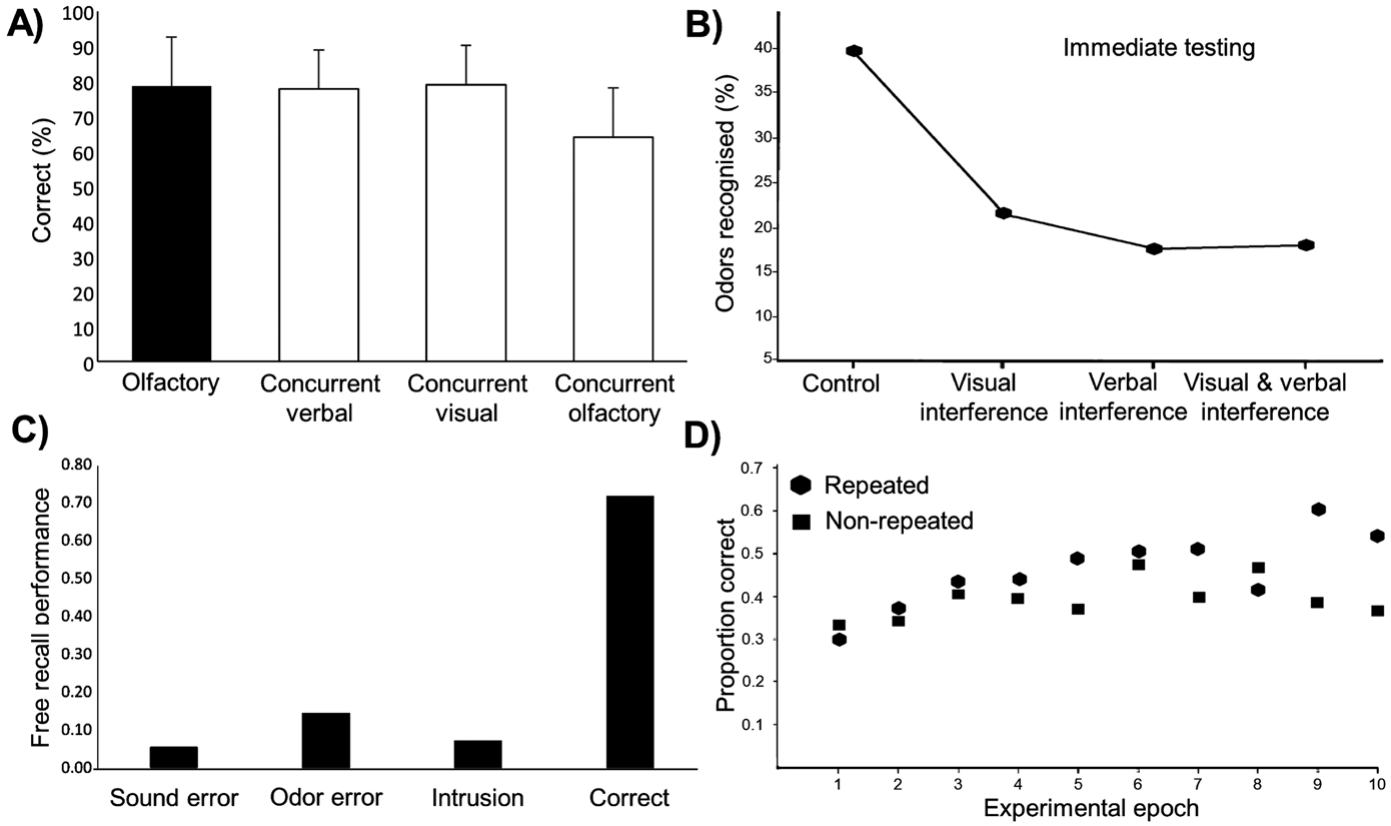
Exemplar Results that Illustrate Benchmarks 7, 9, 11, and 15. *Note*. Panel a) Adapted from results reported in Andrade and Donaldson (2007) for Experiment 2 (mean percent correct scores + standard deviations). Panel b) Figure adapted from recognition testing results reported in Annett et al. (1995). Panel c) was adapted from results reported by White et al. (1998), it shows the proportion of each type of error (item or order) response in a free recall task. Panel d) shows the mean of proportion correct for repeated and nonrepeated sequences of odors, based on results of Figure 2 in Johnson et al. (2013).

Only one OWM study from our review of the literature was relevant for this benchmark (Andrade and Donaldson 2007). That study found that memory for verbal items, such as a list of digits, was decreased when memory for odors was also required, though at a much lower level than the disruption from other verbal material like letters (Andrade and Donaldson 2007, Exp. 1). Memory for a single set of olfactory stimuli is better than for multiple sets of information, particularly if the additional memory set is from the olfactory domain (Andrade and Donaldson 2007, Exp. 2; see Fig. 3a). While this is in keeping with the verbal literature, it is worth noting that these results come from a single publication, so research on this benchmark is sparse. We regard this benchmark as weakly *supported*, and note that more research is needed.

### 3.8. Disruption of memory by processing in the same domain

Verbal memory is disrupted when participants perform a secondary processing task, such as trying to remember a list of words while repeating the word “the” or “racket” while the list is being presented for encoding (e.g. Baddeley et al. 1984; Norris et al. 2018). Disruption of verbal memory can also be achieved when the concurrent articulation is performed between the presentation of the memory list and the test, or in-between the items in the list (e.g. Peterson and Peterson 1959; Vuontela et al. 1999). Examples of such memory-disrupting processing tasks are articulatory suppression (e.g. Baddeley et al. 1975), mental calculations (e.g. Barrouillet et al. 2004), or spatial judgments (e.g. Vergauwe et al. 2009). Typically, the WM performance disruption is most substantial when the items to be remembered and the processing task comes from the same content domain (e.g. Cowan and Morey 2007).

In order to demonstrate disruption of OWM, additional odorants irrelevant to the memory task should be presented between study and test, or in-between the odors to be remembered. Three OWM studies from our review were relevant for this benchmark (Walk and Johns 1984; Zucco 2003; Andrade and Donaldson 2007). Interference is under-researched in OWM, but an early study demonstrated that OWM was impaired if participants verbalized to a distracting odorant that was presented between list acquisition and test (Walk and Johns 1984). Later work showed that when a memory probe was preceded by 2 additional odors, OWM performance was impaired (Andrade and Donaldson 2007). In contrast to these findings, however, Zucco (2003, Experiment 2) failed to demonstrate any olfactory interference from a secondary task involving pleasantness ratings of odors that were interleaved between list presentation and test. At present, the scarce evidence gives *mixed* support for this benchmark and additional research is needed to establish whether OWM behaves similarly to verbal memory in this capacity.

### 3.9. Disruption of memory by processing in another domain

Memory can be disrupted by concurrent processing of distractors that come from a different domain than the memory items, but the impairment to WM is typically less severe (e.g. Chein et al. 2011). Disruptions have been observed between the verbal and visuospatial domains (e.g. Chein et al. 2011), as well as the visual and spatial domains (e.g. Vergauwe et al. 2009). Theoretically, this finding is important for conceptualizing the resources necessary for WM, as it differentiates between competition for shared attention and interference that is based on item representation (e.g. Baddeley and Logie 1999; Oberauer et al. 2012).

Seven OWM studies from our review were relevant for this benchmark (Table 2). The olfactory literature provides mixed results regarding the effects of distractions from nonolfactory domains on memory for odors. Three studies have shown that either verbal (Annett et al. 1995; Miles and Hodder 2005; Experiment 6; Walk and Johns 1984) or visual (Annett et al.,1995) processing tasks can disrupt OWM. For example, Annett and colleagues (1995) found that memory for a 15-odor set was impaired by both visual and verbal suppression tasks (playing a video game or repeating digits presented via headphones) that occurred during the initial presentation of the odorants (see Fig. 3b).

Three other studies showed no olfactory disruption by either verbal, visual, or both types of tasks (Moss et al. 2019; Experiment 2; Zucco 2003; Experiment 2; Andrade and Donaldson 2007; Experiment 2; Miles and Jenkins 2000). In these studies, concurrent articulation during the inter-trial interval of an olfactory n-back task did not reduce recognition sensitivity, even for highly namable odorants (Moss et al. 2019). Neither visual nor auditory suffixes disrupt olfactory serial position recall (Miles and Jenkins 2000). Further, attempts to disrupt odor memory with a mental rotation (visuospatial) task interleaved with the presentation of list items (Moss et al. 2019) were also ineffective in disrupting OWM.

The contradictory results concerning the effects of verbal and visual stimuli on memory for olfactory stimuli have not been adequately addressed. Some specific results have been explained in terms of large individual differences (Habel et al. 2007), task difficulty (Annett and Leslie 1996), or as a byproduct of the specific olfactory stimuli used in a study, with more nameable odorants being more susceptible to memory disruption (Moss et al. 2018); however, the parameters moderating interference of OWM are not clearly delineated and have not been systematically examined. We conclude that the olfactory literature provides *mixed* support for this benchmark, given the conflicting results.

### 3.10. Effect of cognitive load of the processing demand

When people are attempting to perform 2 tasks at one time, the limits of executive function become apparent (e.g. Barrouillet et al. 2007; Ricker and Cowan 2010). Increasing one task’s cognitive load (proportion of time that it captures attention) impairs the ability of another task to garner attention, whether the 2 tasks come from the same content domain (e.g. Barrouillet et al. 2007) or different ones (e.g. Langerock et al. 2014); this results in poorer WM performance. Concurrent tasks that require more executive control (or attention) lead to poorer verbal WM performance (e.g. Liefooghe et al. 2008; Barrouillet et al. 2011), whether the memory task is one of serial recall (e.g. Barrouillet et al. 2007) or recognition of an item’s location (relational recognition; e.g. Ricker and Cowan 2010). This concept is theoretically important, as attentional processing is thought to be a domain-general resource, and one of the mechanisms that can maintain information in WM (Oberauer et al. 2012).

A hint that OWM may be affected by cognitive load of the processing demand comes from a study that shows decreasing memory performance with list length in an OWM span task (MacQueen and Drobes 2017); however, no published article that met our criteria has addressed this benchmark with olfactory stimuli directly. There were a number of articles that did not meet our selection criteria that provided indirect evidence that this benchmark eventually may be supported. For example, a series of tasks conducted as part of a doctoral dissertation demonstrated a reduced accuracy and increased response time in successively more difficult WM tasks [1-back, 2-back, and modified Sternberg task (Sternberg 1966)] presumably due to increasing levels of cognitive load (Radnovich 2008). An unpublished study showed no effect of cognitive load in the form of mental arithmetic in an olfactory 2-back task using either easily verbalized or difficult to name odors (Johnson et al. 2022).

Although the experimental evidence here is limited, if everyday situations are considered, it is well known that people increase food intake while distracted, such as by watching television (e.g. Bellisle et al. 2004; Braude and Stevenson 2014), presumably because they are no longer able to attend to the quantity of their intake or to the food’s flavor. As olfaction is a major component in flavor perception (Mozell et al.,1969), it would seem likely that odors should also show an effect of cognitive load. We regard this benchmark as *unaddressed*, and more evidence is needed.

### 3.11. Phonological similarity

When trying to remember a list of items in order, people very often make confusion errors that reflect phonological similarity rather than visual or meaning-based similarity (Conrad 1964). So, for example, people would be more apt to confuse the letter “E” with “C” (because they sound similar to each other) rather than “F” (which sounds differently). Thus, lists of letters that sound similar (e.g. T, V, B) are more poorly remembered than those that are more distinctive (e.g. L, R, S) in phonology (Conrad and Hull 1964). This effect is observed regardless of whether the verbal item to be remembered is presented visually or auditorily (Peterson and Johnson 1971), which suggests that phonological coding is a less effortful way to code information in WM.

Only one study in our OWM review has directly addressed this benchmark; White et al. (1998) systematically manipulated both the perceptual and phonological similarity of odorants in a serial recall task. Although the results showed effects of phonological similarity, participants exhibited more perceptual errors than phonological ones; so, they were more likely to confuse “thyme” for “sage” rather than for “lime.” This finding suggests that although labeling plays a part in the memory process, perceptual aspects of the stimuli were still a part of the stored memory (see Fig. 3c), and thus this study only gives, at best, weak support for a strict interpretation of this benchmark.

If one were to adopt a more liberal interpretation of this benchmark, however, phonological similarity could be regarded as a specific case of item similarity (e.g. Hurlstone et al. 2014). This liberal interpretation has been applied to visual stimuli such as matrices or faces (Avons and Mason 1999; Smyth et al. 2005), where perceptual similarity affects memory in addition to phonological similarity (e.g. Smyth et al. 2005; Saito et al. 2008). Item similarity effects on memory performance can be readily observed with odorants as well (e.g. Jones et al. 1978). The perceptual similarity between 2 odors affects whether they will be confused in an olfactory recognition memory task (Jones et al. 1978; Mair et al. 1980). Odorants that are more dissimilar from each other are less likely to be confused (Jehl et al. 1994; Wenzel et al. 2021), so olfactory quality has an effect on the number of item errors that participants make in an odor recognition task. Thus, perceptual item similarity clearly plays a part in OWM and also lends some evidence toward this benchmark, so we regard this benchmark as weakly *supported*. Clearly, however, more work should be done to directly address the effects of phonological similarity on WM for olfactory stimuli.

### 3.12. Effects of distinctiveness and of grouping: grouped lists are better recalled

The evidence for this benchmark comes from the examination of performance of verbal serial order memory tasks. Participants typically perform better when the items to be remembered are presented in small groups, distinguished either visuospatially or through a temporal pause, rather than as a single list (e.g. Ryan 1969; Hartley et al. 2016). For example, in the United States, telephone numbers are usually grouped into 2 sets of 3 numbers and a set of 4 numbers to improve accuracy (Wickelgren et al. 1967), and minimize order errors (Ryan 1969). In addition to verbal stimuli, the effect of grouping has been observed with serial memory for spatial and visual stimuli (Hurlstone and Hitch 2015, 2018). This benchmark is as yet *unaddressed* with olfactory stimuli, and it remains an interesting opportunity for investigation.

### 3.13. Prioritization of information in WM: item-switch effects

At any given moment, only a limited number of WM items can be represented in a readily accessible state (Baddeley 1986; Cowan 1995). Cognitive tasks involving updating usually require participants to attend selectively to a subset of the contents of WM (e.g. Garavan 1998; Oberauer 2003b). Such tasks include keeping separate running counts of the number of triangles and rectangles that appear on a screen (one by one). This task requires that people temporarily prioritize individual items within WM by updating each counter, an activity that comes at a cost to the other information stored there, namely the total for the other geometric shape (e.g. Garavan 1998). Items maintained in the attentional focus are better remembered and retrieved more quickly than items that are not within the current focus (e.g. Garavan 1998; Oberauer 2003b); thus, people are faster to respond to a triangle after a triangle than after a rectangle (Garavan 1998), as there is a cost to switching the attentional focus (e.g. Oberauer 2006). This finding has been found in verbal (e.g. Oberauer 2003b), visual (e.g. Garavan 1998), and spatial memory (e.g. Hedge and Leonards 2013).

No OWM study that we reviewed directly addressed this benchmark. Indirect evidence was provided by Johnson and Allen (2022) in a 3-experiment paper that examined learned associations between odors and colors. Although one of the experiments (Experiment 2) showed that participants were able to prioritize the first learned pairing of the color and odors at the expense of later associations, the color names were retrieved from memory, whereas olfactory information was not retrieved, thus making this research a less direct assessment of the benchmark. There is, to our knowledge, no experiment that explicitly examined this issue solely with olfactory stimuli, so it is unknown if the benchmark extends to olfaction; the benchmark is *unaddressed*.

### 3.14. Effects of chunking

Chunking is the improved retention of information that contains patterns (chunks) that the participant has encountered previously, particularly those that carry some meaning or make use of stored knowledge. For example, a series of numbers that includes something that includes your zip code is probably easier to recall than others. In chunking, people make use of long-term memory (LTM) in order to apparently increase the capacity of WM for verbal (Ericsson et al. 1980) or visual stimuli (Chase and Simon 1973). The apparent WM increases are facilitated by experience (Ericsson and Kintsch 1995) and expertise (e.g. Ericsson et al. 1980) specific to the type of material that has been mastered by the individual; other types of materials show little memory advantage (Chase and Simon 1973; Gobet and Simon 1996).

No OWM studies from our review have directly assessed this benchmark. Researchers have likely considered the phenomena, as chunking has been suggested as a possible memory mechanism that might be of use to perfumers (Dalton 1996). Grouping the scents into perceptual categories as a memory aid would be considerably easier with expertise, and the mere observation that experts are better than novices at olfactory memory tasks (Valentin et al. 2007, 2011) suggests that those with experience use some type of organizational strategy (Parr et al. 2004). This strategy could simply be increased efficiency in the usage of labeling (Melcher and Schooler 1996; Cessna and Frank 2013), but at least some studies argue against that as an explanation (Croijmans et al. 2021), so chunking remains a possible memory strategy. Direct evidence for chunking with olfactory stimuli, however, is lacking, and though it might be explored in future research, this benchmark remains *unaddressed*.

### 3.15. Hebb repetition effect

Hebb (1961) had people perform an immediate serial recall task where unbeknownst to them, the same list was used on multiple trials. He found that recall of the repeated list improved as a function of the number of repetitions; essentially, this is another instance of LTM performance improving apparent WM performance. The Hebb repetition effect has been demonstrated with both verbal (e.g. Hebb 1961) and visuospatial (e.g. Couture and Tremblay 2006; Ueda et al. 2023) stimuli.

Only one OWM study was relevant for this benchmark (Table 2); a direct test of the Hebb repetition effect confirmed that the effect was present when using olfactory stimuli (Johnson et al. 2013), with significantly improved memory performance for odor sequences that were repeated every third trial over ones that were not (see Fig. 3d). Indirect evidence to support this benchmark comes from the finding that odors that have been presented to participants repeatedly (several exposures) typically result in greater OWM memory performance (Dade et al. 2002; Nguyen et al. 2012), though this manipulation also results in better and faster identification (Olsson 1999; Broman et al. 2001) which could foster recoding for verbal memory. We regard this benchmark to be weakly *supported* due to the sparse evidence. Additional research is important to verify the result.

### 3.16. Positive manifold

Positive manifold is the idea that the variables that relate to performance on WM tasks correlate positively with each other despite variations in tasks and materials because they all relate to a shared underlying WM factor. For example, scores on reading span tasks are not only correlated across verbal and visuospatial stimuli, but they are also related to scores on other types of WM tasks, such as a listening span task (e.g. Daneman and Carpenter 1980). The concept of positive manifold supports the construct validity of WM and is important to ideas of inter-individual variations that may exist in WM capacity (Unsworth et al. 2014).

Three OWM studies in our evaluation were relevant for this benchmark (Danthiir et al. 2001; Doty et al. 2015; MacQueen and Drobes 2017). These studies all evaluated their participants with both olfactory and nonolfactory WM tests; results of each of the studies showed some level of positive correlation between some olfactory tests (olfactory swaps; olfactory span task; odor memory/discrimination test) and similar tests in other modalities (visual and verbal), which could be representative of WM. However, in each case only some (not all) of the assessments of WM in other modalities demonstrated a relationship with OWM. To better understand these relationships, an ambitious study by Danthiir et al. (2001) analyzed their data using a factor-analytic method to assess the latent cognitive abilities that may have affected performance on olfactory and nonolfactory tests (Danthiir et al. 2001). Results gave support for an underlying “cognitive olfactory factor,” as different cognitive olfactory tests were most strongly associated with each other, rather than with their nonolfactory counterparts. Importantly, the authors controlled for varying olfactory abilities among participants, which would otherwise have influenced all olfactory cognitive tests and biased the outcome. The result suggests that OWM does not seem to be part of the same positive manifold as nonolfactory WM tests, but rather relies on its own cognitive domain.

The olfactory cognitive factor posited by Danthiir and colleagues (2001) built on a substantial literature that shows positive correlations among olfactory cognitive tests. For example, scores on an olfactory version of a Sternberg task were correlated with their performance on both olfactory 1-back and 2-back tasks (Radnovich 2008). OWM span varies widely between individuals and correlates positively with olfactory discrimination ability (Dacremont and Valentin 2004). It should be noted that this relationship is not restricted to olfactory cognitive tasks; many olfactory tasks correlate positively with each other (Doty et al. 1994).

Besides OWM tests correlating positively with other tests of olfactory cognitive ability, a recent systematic review (Ramaswamy and Schofield 2022) concluded that there was a strong association between cognitive function and olfaction; however, in the articles that formed the basis of the review, olfaction was mostly assessed with odor identification, which involves semantic cognition (e.g. Raj et al. 2023). Despite the strands of evidence suggesting that olfactory tests are positively associated with each other, as well as with visual cognitive tests, there is no evidence for a clustering of olfactory and nonolfactory WM tasks specifically (e.g. as could be established by latent factor analysis after controlling for olfactory sensory ability) which would meet the positive manifold benchmark.

In sum, we regard the evidence to date as not supporting the benchmark in the olfactory domain. Olfactory WM capacity can not be viewed as a reflection of “general” WM capacity observed for visual or auditory materials. Instead, performance may be dependent on an olfactory-specific cognitive factor, and thus, the benchmark is *unsupported*.

### 3.17. Correlation between WM and attention indicators

Attention plays an important role in most models of WM (e.g. Baddeley 1986; Cowan 2008) and WM capacity is strongly related to indicators of controlled attention (e.g. Kane and Engle 2003). Attentional indicators are the rules, instructions, or goals that place minimal demands on memory in terms of item storage but must still be maintained in order to complete a WM task. For example, the task instructions in a Stroop task would be considered an attentional indicator (Kane and Engle 2003); the task requires maintenance of the task goal, and resolution of response competition, but there is not a memory load per se, because none of the words needs to be remembered (Stroop 1935). Three types of attentional indicators are particularly robust in their relationship with WM capacity: (i) The Stroop task has a higher error rate when performed by people with lower WM capacity, as assessed by a complex span task (Kane and Engle 2003; Meier and Kane 2013). (ii) The antisaccade task is executed with a higher level of accuracy by people with greater WM span (Hallett 1978), indicating they are better at resolving the competition between a goal and a reflex (Kane et al. 2001; Meier et al. 2018). (iii) A lower level of mind-wandering is associated with a higher WM capacity (e.g. McVay and Kane 2009).

No OWM study that we reviewed was directly relevant for this benchmark. Indirect evidence hints at the idea that attentional indicators may be pertinent to OWM, as performance on odor discrimination and identification tasks can be improved by minimizing memory demands of the instructions and goals of the tasks (Zucco et al. 2014). Odor processing can be influenced by distraction (Forster and Spence 2018) and odors can themselves be distracting (Unsworth and McMillan 2017; Hörberg et al. 2020). When attentional lapses (similar to mind-wandering) were indicated by EEG activity in the delay in-between an odor cue (e.g. pear odor) and a word target (e.g. “pear”), participants were slower to decide whether they match or not (Olofsson et al. 2018), suggesting difficulties in processing. Although odorants have been used to examine cross-modal Stroop effects (Pauli et al. 1999; White and Prescott 2007; Finkelmeyer et al. 2010; Xu et al. 2020), WM capacity has not been evaluated in those studies. This benchmark remains *unaddressed* and is a target area for future olfactory research.

### 3.18. Correlation of WM with fluid intelligence

Intelligence varies across individuals and consists of crystalized knowledge (which is accumulated over time and depends on LTM) as well as fluid intelligence (Cattell 1963). General fluid intelligence is the ability to solve problems abstractly, and reason, without necessarily using past knowledge. Verbal WM task performance is strongly correlated with measures of general fluid intelligence (Kane et al. 2005; Oberauer et al. 2005).

For olfactory research to support this benchmark, a relationship between performance on a general test of fluid intelligence and OWM tasks would be required. Only one OWM study in our review evaluated a relationship of this type and thus was directly relevant to this benchmark; Danthiir et al (2001) tested more than 100 participants on 12 psychometric tests, 4 putative cognitive olfactory tasks, and 1 olfactory discrimination task. Two different OWM tasks showed correlations with tests of fluid intelligences, both verbal (letter swaps) and nonverbal (Raven’s Progressive Matrices), with coefficients ranging from 0.26 to 0.49. A factor analysis showed that one olfactory test (olfactory swaps) loaded significantly on the same factor as the other tests of general fluid intelligence. This research gives *support* for this benchmark in the olfactory domain, although weakly, as it depends only on one experimental result.

### 3.19. Dissociable neural substrates of different content domains

When different types of information are retained in WM, different brain regions are engaged. Some models of WM emphasize such differences as manifestations of how content representations in different WM subsystems are engaged in WM processes (e.g. Baddeley 1986). Generally, representation of visuospatial information is separated, such that spatial WM is represented in dorsal areas, including the superior frontal sulcus and parietal cortex (e.g. Courtney et al. 1998), while visual object WM is represented in ventral areas of cortex including the inferior frontal gyrus and temporal cortex (e.g. Ungerleider et al. 1998). Furthermore, a considerable amount of human neuroimaging evidence indicates that verbal WM leads to a cortical engagement of left inferior frontal and perisylvian areas (e.g. Awh et al. 1996; Smith et al. 1996; Rottschy et al. 2012).

Our review found that 5 OWM publications support this benchmark. Three of these articles by Dade and colleagues (Dade et al. 1998, 2001, 2002) used, PET imaging and the results supported the notion that the cortical substrates for OWM appear at least partly unique to odors. A short-delay odor recognition task indicated brain activity in the right dorsolateral prefrontal cortex (Dade et al. 1998). Although WM tasks such as n-back may elicit similar brain activation for both visual and olfactory stimuli (dorsolateral and ventrolateral frontal cortices), sensory-specific activation may also be observed, as only the visual stimuli were associated with activity in the left superior parietal area (Dade et al. 2001).

The 2 other studies identified in our search also offer support for this benchmark. A delayed match to sample task garnered differential brain activity for nameable odors (inferior frontal gyrus) compared to hard-to-name odors (frontal piriform cortex) and auditory tones, indicating that separate brain regions are responsible for processing these different types of stimuli (Zelano et al. 2009). Another paper showed that ERP measurements conducted during an olfactory change-detection paradigm had a pattern of frontotemporal activation that was parallel, but separate from activity observed in other sensory modalities, though the 2 types of activation were not directly compared in that study (Lenk et al. 2014).

Other olfactory studies that did not meet our review criteria have indirectly supported this benchmark through the notion of olfactory-specific content domain in various short-term memory tasks. PET studies indicated that olfactory short-term memory in a 1-back task (Savic et al. 2000) or a paired discrimination task (Kareken et al. 2003) involved areas such as the right orbitofrontal cortex, hippocampus, subiculum, caudate, thalamus, cerebellum, frontal operculum, cingulate, and the left insula. Further evidence comes from cue-target matching experiments that may tax WM (Olofsson et al. 2014). When target words are matched to a recent perceptual odor cue, incongruent targets (e.g. “lemon” after smelling a rose) re-activates olfactory-associated cortical regions and generates an ERP waveform with a different topology, compared to when the same targets are matched to a preceding picture (e.g. a picture of a lemon; Olofsson et al. 2014).

The evidence (direct as well as indirect) strongly suggests that OWM is processed by neural WM substrates that are separate from other types of content domains. In this way, the distinctiveness of olfaction is to be expected, as other domains have their own WM signatures. The benchmark is *supported* by the olfactory evidence.

### 3.20. Preserved WM in amnesia

WM is distinct from LTM, as shown by evidence from neuropsychological case studies (e.g. Baddeley and Warrington 1970, 2017). Amnesic patients typically show a double dissociation in which some patients may have WM deficits without LTM deficits, if they have lesions of the frontal and perisylvian cortex, while patients with damage to the medial temporal lobe (MTL), such as hippocampus or amygdala, show instead the opposite pattern of memory difficulties of LTM deficits with intact WM (Scoville and Milner 1957; Baddeley and Warrington 1970; Cave and Squire 1992). Although such double dissociations have been regarded as providing definitive evidence, recent work has suggested that some level of WM memory impairment is observed in patients with lesions in MTL (e.g. Finke et al. 2008), though the deficits are not as severe as those in LTM (e.g. Jeneson and Squire 2011).

Two OWM studies from our review were relevant for this benchmark (Eskenazi et al. 1983; Levy et al. 2003). Eskenazi and colleagues (1983) studied the performance of patients with unilateral temporal lobe excisions on several olfactory tests as compared to controls. The researchers found that while patients had normal odor thresholds, they were impaired on olfactory cognitive and short-term memory tests, including OWM. Levy and colleagues (Levy et al. 2003) studied amnestic patients with hippocampal damage on an odor span task that assessed odor WM. The patients were impaired on the task, relative to a healthy control group. However, the patients were also impaired on visual versions of the task where common objects or abstract images were to be remembered. Other research suggests that amnesics with hippocampal lesions are able to retain some olfactory information over short periods of time but are unable to form longer-term memories (Levy et al. 2004). Patient H.M., possibly the most famous amnesic with MTL damage, showed an impaired ability to identify and discriminate odor qualities in a same-different task, though his ability to detect odors and to discriminate their intensity levels was preserved (Eichenbaum et al. 1983). To summarize, although amnesia due to MTL damage is associated with poor OWM, we do not have evidence for preserved OWM but impaired LTM. Because we do not have both halves of the double dissociation, we regard this benchmark as having *mixed* evidence, and further investigation is warranted.

### 3.21. Measures of neural activity track amount of information in WM

WM is thought to distinguish itself from LTM in terms of the transience of the associated neural code (Goldman-Rakic 1995), which seems to be conveyed through neural firing (Fuster and Alexander 1971; Kubota and Niki 1971). Estimates of the number of items retained in a WM task seem to be tightly correlated to fMRI and EEG activity, particularly for visual information (Todd and Marois 2005; Luria et al. 2016); evidence for this benchmark in verbal and spatial WM is less clear. We have found no published attempts to associate OWM activity or capacity in this way. This benchmark thus remains *unaddressed*, for future OWM research.

## 4. Conclusion

Theories of WM often assume domain-general applicability (e.g. Oberauer et al. 2018b). Olfaction provides an interesting case for the generalizability of higher cognitive functions, as olfaction shows some behavioral differences from visual/verbal stimuli by being poorly verbalized and not localized in space (Herz and Engen 1996; Olofsson and Gottfried 2015). Odors are also intrinsically different from the visual, spatial, verbal, and auditory content domains that are most studied in relation to WM in that the presentation of odors must proceed serially and cannot be perceived simultaneously (as verbal or visual stimuli can be) without having a tendency to be perceived as a mixture, or a new odor (Jinks and Laing 1999). This implies that almost every olfactory daily experience, such as distinguishing one smell from another, may engage WM. Further, it also implies that most experiments potentially engage WM, making memory central to olfactory processing (Wilson and Stevenson 2006; Stevenson and Wilson 2007).

**Fig. 4.**
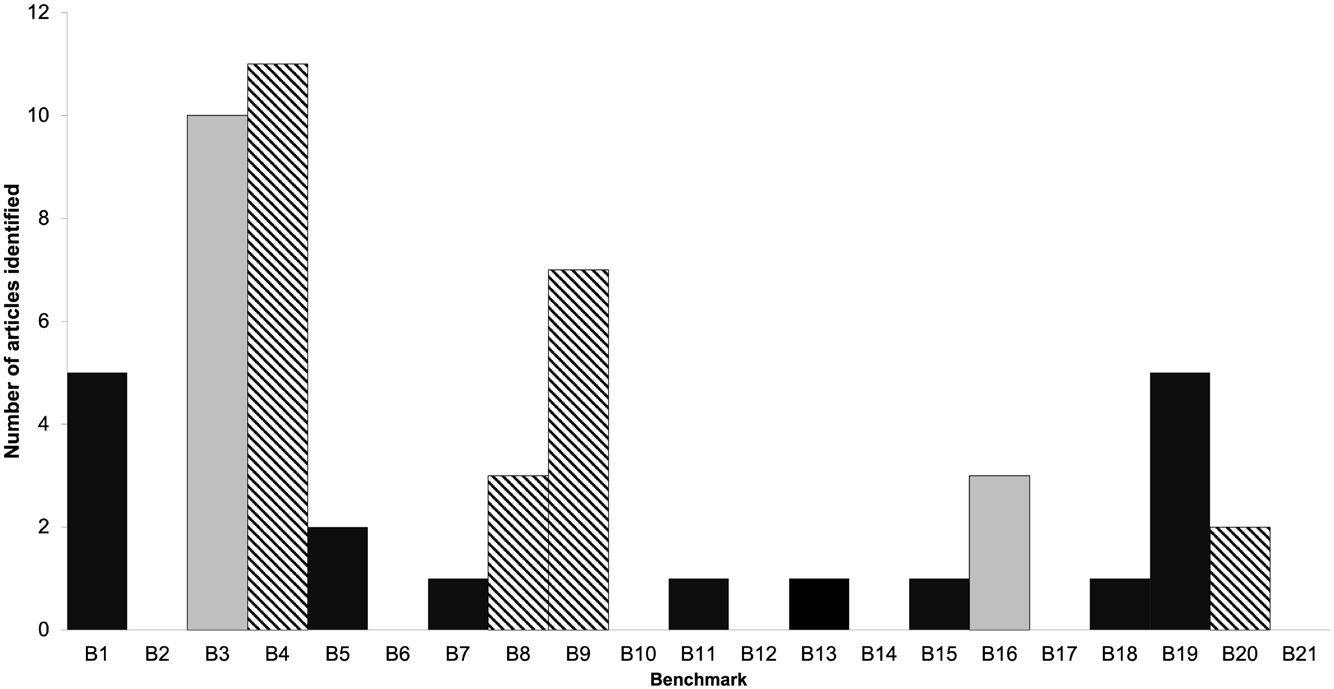
Summary of Systematic Review Results for 21 WM Benchmarks. *Note.* Summary of results from an evaluation of the 21 “A” WM benchmarks outlined by Oberauer et al. (2018a) with the olfactory literature, numbered as detailed in the text. Supported benchmarks are coded in black, and unsupported benchmarks are coded in gray. Mixed support was coded in hash lines, and benchmarks for which there is an absence of research have no bars.

The present paper addressed a fundamental question, which is whether the olfactory system operates in a way that is consistent with WM “A-level” benchmarks that have been well-established with verbal, visual, and spatial memory; we did not examine “B-level” or “C-level” benchmarks in the current paper. Our results, based on a systematic review of the evidence accumulated during the last 50 yr of research, are summarized in Fig. 4. Of the 21 “A-level” WM benchmarks considered, we could confirm 7 as supported in research with olfactory stimuli, while 2 were distinctly different for odors as compared to other senses. It is also notable that for more than half (12) of the relevant benchmarks, the evidence was either absent (8) or mixed (4), preventing a conclusion. Three points are abundantly clear from an examination of this evidence. Firstly, OWM shares many properties of WM in other senses. Secondly, OWM differs in some key aspects from other senses. Thirdly, the gaps in the literature indicate that OWM is a ripe area for future research that may help elucidate the structure of WM (see Fig. 4). Below, we discuss the implications of the evidence.

Many benchmarks that were identified for verbal and visuospatial memory (Oberauer et al. 2018a) generalize to olfaction and suggest that OWM is in many ways similar in quality to other types of WM. In general, the evidence from the OWM literature reviewed here mostly provides at least preliminary support for the assumption of generalizability across senses and adds to the larger set of empirical findings that form the basis of domain-general theoretical accounts of WM. OWM, like that of other senses, has capacity limitations that become apparent at larger set-sizes. Odors held in WM are often confused with each other, similarly to what is observed in visual and auditory materials. OWM is disrupted by concurrent tasks, particularly if those tasks involve odorants. The finding that fluid intelligence is related to OWM suggests that some limits on the capacity of WM may be related to domain-general individual differences. The facilitation of WM performance by LTM, as shown in the Hebb repetition effect, is also similar across stimulus domains, indicating that the underlying mechanism is quite general. Further, like the verbal, spatial, and visual systems, the neural substrates for OWM are separable from those of other sensory systems.

There are also notable differences between WM performance with olfaction as compared to other types of stimuli. When odors are held in WM along with visual or auditory stimuli, performance is decreased, but less so compared to the distractions (either from other verbal or olfactory stimuli) observed among the visual-auditory senses. The high positive correlations among cognitive and WM abilities in visual and auditory systems are not present in OWM. Olfactory cognitive tests show a positive manifold with each other, not primarily with their visual or auditory counterparts. Olfaction is, in these cases, essentially an outlier in comparison to verbal, visual, or spatial WM. These discrepancies are of particular theoretical relevance, as they may suggest an increased level of domain-specificity in olfactory cognition. We propose that whereas visual and auditory cognition are well-integrated in WM operations, olfaction may constitute its own perceptual-cognitive system. This olfactory cognitive system is instead well-integrated with episodic encoding systems, as is reflected in the high level of connectivity between olfactory cortices and the hippocampus (Zhou et al. 2021). Presumably, visual and auditory WM are more well-integrated with each other than they are with olfaction due to the fact that visual and auditory systems often operate in tandem in everyday life, when we are seamlessly alternating between WM engagements in reading, talking, verbalizing, and writing. Olfaction has instead been described as a mostly nonverbal cognitive domain (Olofsson and Gottfried 2015), that associates sensations with inner states, motivations, and emotions (Stevenson 2010).

What can the evidence from olfactory research add to the debate about the general structure of WM? The purpose of the present paper was to review the evidence for OWM benchmarks, rather than to develop a new theoretical framework. We do believe, however, that the evidence reviewed here is of theoretical importance, as models of WM vary considerably, from denying the need for any distinction between short and LTM (Nairne 2002; Macken et al. 2015) to proposing a difference between very active and less active retrieval cues (Unsworth and Engle 2007) or memories (Oberauer 2013; Cowan 2017), to specialized storage buffers or mechanisms of retrieval (Logie 2011; Baddeley 2012; Barrouillet and Camos 2015). Most of these theories do not include differences between sensory modalities other than in a cursory fashion. Previous attempts have been made to fit findings from OWM research into wider theoretical frameworks (e.g. Herz and Engen 1996; White 1998, 2009; Wilson and Stevenson 2006; Baddeley 2012); findings from specific studies have been frequently interpreted in terms of the addition of a proposed distinctive olfactory storage buffer [e.g. the “censer” by White (1997); olfactory “flacon” by Zelano et al. (2009); and the “palette” by Dade et al. (2001)] to a multistore model, but also could be explained with an olfactory-specific unitary-store memory model (e.g. Wilson and Stevenson 2006). In the absence of theoretical convergence, benchmarks provide reliable communal metrics of the WM phenomena. We hope that OWM experiments, guided by the evidence provided here, will help theorists to develop WM frameworks with a truly domain-general scope.

The results of the present research review might facilitate theoretical development in odor imagery. Some researchers have argued that people mostly lack the ability to imagine smells (Engen 1991). However, there is some empirical support for human odor imagery, including that people can faithfully recreate odor quality similarities (Pierzchajlo et al. 2025) or intensities (Algom and Cain 1991) from memory, that sniffing behavior serves a facilitating function in odor imagery just like eye-movements in visual imagery (Bensafi et al. 2003), and that mental images of odors might drive food cravings and intake (Perszyk et al. 2023). Since OWM processes might be used in odor imagery tasks, the present review might guide researchers investigating this contested topic.

It is worth noting some of the limitations of OWM evidence. First, unlike WM for verbal material, participants cannot repeat the stimuli (Stevenson and Attuquayefio 2013), so WM measurements are restricted to either recognition tasks or cross-modality response tasks such as reporting the name of a smell. This is an advantage of verbal relative to olfactory WM assessments. Second, details of study procedures, such as the time to respond, or details of the types of errors in a memory task, are not always present in the OWM literature, though such details would assist in integrating olfactory research with the wider memory literature. In addition, many existent OWM studies do not distinguish between tasks involving novel odors and those that involve familiar odors; highly familiar (and presumably nameable) odors may well tap into different aspects of WM (Hasselmo and Stern 2006), possibly through the recruitment of other types of WM, perhaps through verbal recoding, or maybe through LTM. Though odorants are notoriously difficult to label effectively (e.g. Cain and Potts 1996), verbal re-coding of odorants may strongly influence OWM performance by providing verbal associations (Herz and Engen. 1996; Zucco 2007; Moss et al. 2018) and OWM performance for namable odorants is more subject to disruption from verbalization (Moss et al. 2018). This suggests that on occasion, what is apparently OWM may be a product of remembering the label rather than the odor per se; Table S2 lists the stimuli that were reported for each of the articles selected by our review. Third, as became clear during this work, some OWM research is conducted outside of the 21 benchmark topics. This is not strictly a limitation, as it might reflect other valid priorities, such as alignment with neuroscientific paradigms and theories, rather than human cognition. But in this context, it might lead to a weaker integration of olfaction in the human WM literature. We hope that OWM research develops along multiple paths, ranging from its possible roles in everyday olfactory experiences (e.g. novelty, anticipation, and cooking of food) to clinical olfactory disorders and complaints (e.g. parosmia, phantosmia, and multiple chemical sensitivity).

For many years, the study of cognition associated with olfactory stimuli has lagged behind the evaluation of verbal and visual stimuli. Several benchmarks are unaddressed, scarcely addressed, or provide contradictory (mixed) outcomes in OWM. The limited nature of the research has led to many questions left unanswered about OWM; for example, there is theoretical debate as to whether a short-term OWM store is an active WM (e.g. Dade et al. 2001; Jönsson et al. 2011; Moss et al. 2019) or merely a passive store (Stevenson 2011; Zucco and Job 2012; Wenzel et al. 2021). We hope that our overview will provide guidance for future work that may fill in gaps in the literature and potentially resolve such conflicts; there are many opportunities for discovery. Future olfactory research into unaddressed benchmarks with sufficiently powered, preregistered studies will clarify the nature of OWM.

The results from our examination of well-established WM benchmarks strengthen the notion that overall, OWM is similar to WM in “higher” senses, such as audition or vision. We have also shown that OWM has some unique properties, mainly regarding how OWM interacts with other processes, that warrant further investigation. We hope that these properties, coupled with the general scarcity of research, will encourage future exploration into the way that higher mental abilities are enabled by “the cognitive nose.”

## Supplementary Material

bjaf008_suppl_Supplementary_Table_S1

bjaf008_suppl_Supplementary_Table_S2

bjaf008_suppl_Supplementary_Table_S3

## Data Availability

The data consist of research articles that are already in the public domain.
