# Supplementary material for: A cognitive nose? Evaluating working memory benchmarks in the olfactory domain
Source: Chem Senses. 2025 Mar 10;50:bjaf008. doi: 10.1093/chemse/bjaf008 (PMC11985691; doi:10.1093/chemse/bjaf008)
Supplement: bjaf008_suppl_Supplementary_Table_S1 [file bjaf008_suppl_supplementary_table_s1.docx]

**Supplementary Table 1**

*Critical Appraisal of the Revised Studies*

|  | **Author (year)** | **1. Were the criteria for inclusion in the sample clearly defined?** | **2. Were the study subjects and the setting described in detail?** | **3. Was the exposure measured in a valid and reliable way?** | **4. Were objective, standard criteria used for measurement of the condition?** | **5. Were confounding factors identified?** | **6. Were strategies to deal with confounding factors stated?** | **7. Were the outcomes measured in a valid and reliable way?** | **8. Was appropriate statistical analysis used?** | **Overall appraisal** |
| --- | --- | --- | --- | --- | --- | --- | --- | --- | --- | --- |
| 1 | Engen et al. (1973) | Yes | No | Yes | Yes | Yes | Yes | Yes | Yes | 7 |
| 2 | Jones et al. (1975) | Yes | Yes | Yes | Yes | Yes | Yes | Yes | Yes | 8 |
| 3 | Jones et al. (1978) | Yes | No | Yes | Yes | Yes | Yes | Yes | Yes | 7 |
| 4 | Mair et al. (1980) | No | Yes | Yes | Yes | Yes | Yes | Yes | Yes | 7 |
| 5 | Eskenazi et al. (1983) | Yes | Yes | Yes | Yes | Yes | Yes | Yes | Yes | 8 |
| 6 | Walk & Jones (1984) | Yes | Yes | Yes | Yes | Yes | Yes | Yes | Yes | 8 |
| 7 | Murphy et al. (1991) | Yes | No | Yes | Yes | Yes | Yes | Yes | Yes | 7 |
| 8 | Doty et al. (1994) | Yes | Yes | Yes | Yes | Yes | Yes | Yes | Yes | 8 |
| 9 | Jehl et al. (1994) | Yes | Yes | Yes | Yes | Yes | Yes | Yes | Yes | 8 |
| 10 | Annett et al. (1995) | Yes | No | Yes | Yes | Yes | Yes | Yes | Yes | 7 |
| 11 | Annett & Lorimer (1995) | Yes | No | Yes | Yes | Yes | Yes | Yes | Yes | 7 |
| 12 | Bromley & Doty (1995) | Yes | Yes | Yes | Yes | Yes | Yes | Yes | Yes | 8 |
| 13 | Doty et al. (1995) | Yes | Yes | Yes | Yes | Yes | Yes | Yes | Yes | 8 |
| 14 | White & Treisman (1997) | Yes | No | Yes | Yes | Yes | Yes | Yes | Yes | 7 |
| 15 | Dade et al. (1998) | Yes | No | Yes | Yes | Yes | Yes | Yes | Yes | 7 |
| 16 | White et al. (1998) | Yes | Yes | Yes | Yes | Yes | Yes | Yes | Yes | 8 |
| 17 | Miles & Jenkins (2000) | Yes | No | Yes | Yes | Yes | Yes | Yes | Yes | 8 |
| 18 | Reed (2000) | No | Yes | Yes | Yes | Yes | Yes | Yes | Yes | 7 |
| 19 | Dade et al. (2001) | Yes | Yes | Yes | Yes | Yes | Yes | Yes | Yes | 8 |
| 20 | Danthiir et al. (2001) | Yes | Yes | Yes | Yes | Yes | Yes | Yes | Yes | 8 |
| 21 | Dade et al. (2002) | Yes | Yes | Yes | Yes | Yes | Yes | Yes | Yes | 8 |
| 22 | Choudhury, et al. (2003) | Yes | Yes | Yes | Yes | Yes | Yes | Yes | Yes | 8 |
| 23 | Levy et al. (2003) | Yes | Yes | Yes | Yes | Yes | Yes | Yes | Yes | 8 |
| 24 | Zucco et. al (2003) | Yes | No | Yes | Yes | Yes | Yes | Yes | Yes | 7 |
| 25 | Dacremont & Valentinl (2004) | No | Yes | Yes | Yes | Yes | Yes | Yes | Yes | 7 |
| 26 | Miles et al. (2005) | No | Yes | Yes | Yes | Yes | Yes | Yes | Yes | 8 |
| 27 | Andrade & Donaldson. (2007) | Yes | Yes | Yes | Yes | Yes | Yes | Yes | Yes | 8 |
| 28 | Johnson & Miles (2007) | Yes | Yes | Yes | Yes | Yes | Yes | Yes | Yes | 8 |
| 29 | Doty et al. (2008) | Yes | Yes | Yes | Yes | Yes | Yes | Yes | Yes | 8 |
| 30 | Yeshurun et al. (2008) | Yes | Yes | Yes | Yes | Yes | Yes | Yes | Yes | 8 |
| 31 | Johnson & Miles (2009) | Yes | Yes | Yes | Yes | Yes | Yes | Yes | Yes | 8 |
| 32 | Zelano et al. (2009) | Yes | Yes | Yes | Yes | Yes | Yes | Yes | Yes | 8 |
| 33 | Jönsson et al. (2011) | Yes | Yes | Yes | Yes | Yes | Yes | Yes | Yes | 8 |
| 34 | Valentin et al. (2011) | Yes | No | Yes | Yes | Yes | Yes | Yes | Yes | 7 |
| 35 | Zucco, et al. (2011) | Yes | Yes | Yes | Yes | Yes | Yes | Yes | Yes | 8 |
| 36 | Johnson, et al. (2013) | No | Yes | Yes | Yes | Yes | Yes | Yes | Yes | 7 |
| 37 | Lenk et al. (2014) | Yes | Yes | Yes | Yes | Yes | Yes | Yes | Yes | 8 |
| 38 | Doty et al. (2015) | Yes | Yes | Yes | Yes | Yes | Yes | Yes | Yes | 8 |
| 39 | MacQueen & Drobes (2017) | Yes | Yes | Yes | Yes | Yes | Yes | Yes | Yes | 8 |
| 40 | Moss et al. (2018) | Yes | Yes | Yes | Yes | Yes | Yes | Yes | Yes | 8 |
| 41 | Moss et al. (2019) | Yes | Yes | Yes | Yes | Yes | Yes | Yes | Yes | 8 |
| 42 | Wenzel et al. (2021) | Yes | Yes | Yes | Yes | Yes | Yes | Yes | Yes | 8 |
| 43 | Yang et al. (2021) | Yes | Yes | Yes | Yes | Yes | Yes | Yes | Yes | 8 |
| 44 | Johnson & Allen (2022) | Yes | Yes | Yes | Yes | Yes | Yes | Yes | Yes | 8 |
