# Supplementary material for: A cognitive nose? Evaluating working memory benchmarks in the olfactory domain
Source: Chem Senses. 2025 Mar 10;50:bjaf008. doi: 10.1093/chemse/bjaf008 (PMC11985691; doi:10.1093/chemse/bjaf008)
Supplement: bjaf008_suppl_Supplementary_Table_S2 [file bjaf008_suppl_supplementary_table_s2.docx]

**Supplementary Table 2**

*Odor Stimuli for Each Publication*

| **Authors**  **(year of publication)** | **Odor Stimuli** |
| --- | --- |
| Engen et al. (1973) | A set of 100 diverse odorants formed a presentation library, from which a random sample was drawn for each participant. The full list of odorants was not disclosed, but the library included household odorants that were familiar, like garlic and vanilla, as well as chemical compounds like alcohols, and perfumes such as oils. |
| Jones et al. (1975) | A set of 10 food and spice odorant essences chosen to be relatively unfamiliar to participants were selected for presentation: allspice, black walnut, rosemary and sage; and chemical compounds (benzaldehyde; 1-butano, butyl acetate, 1-decanol, d-p menthe-1, 8 diene, and terpinyl acetate. |
| Jones et al. (1978) | Eleven common dried herbs or seasonings packaged by Schilling were in this study: basil (Labiatae Ocumum basilicum), bay (Lauineae Umbellularia californica), celery iUmbeiliferae Apium graveolens), marjoram (Labiatae Origanum majorana), mint (Labiatae Mentha spicatay, oregano (Labiatae Origanum vulgare), parsley (Umbellifeae Petroselinum arispum), rosemary (Labiatae Rosemarinus officinalis), sage (Labiatae Salvia officinalis), tarragon (Compositae Artemisia dracunoulus), and thyme (Labiatae Thymus vulgaris). |
| Mair et al. (1980) | Both similar and dissimilar odorant pairs were formed from combinations of the following odorants: Butyric acid, Oil of cloves, Rapeseed oil, Methyl salicylate", Heptanol, Novoviol, Benzyl butyrate, Lialool, Isopropanol, Vanilla extract, Aldehyde C-14, Cinnamyl propionate", Amyl propionate, Citronellyl butyrate, Aldehyde C-16", Geranyl butyrate, Citral rectified, Acetate C-8, Eugenol", Allyl sulfide, Benzaldehyde, Oil of neuroli, Phenylethyl alcohol, Musk, Anethol, Amyl acetate, Aldehyde C-10, Butanol, Oamma-dodecalactone, Cinnamyl butyrate, Allyl caproate", Citronellyl acetate, Aldehyde 430, Geranyl propionate, Lemon extract and Acetate C-10. |
| Eskenazi et al. (1983) | The approximately equally moderately intense odorants were solutions made from adding odorless diethyl phthalate to L-carvone, ethyl-n-butylamine, 2.3-pentanedione, berualdehyde, and pyridine. |
| Walk & Johns (1984) | The odorants were 36 common food substances that were chosen for their familiarity: barbecue sauce, ketchup, seafood sauce, soya, Tabasco, Worcestershire, propylene glycol, citral, orange extract, pineapple extract, B-ionine, strawberry aldehyde, basil, cloves, nutmeg, oregano, rosemary, thyme, chili powder, curry powder, ginger, onion powder, black pepper, paprika, butter rum extract, vanilla extract, butterscotch extract, chocolate extract, maple extract, mocha, beet, carrot, green pepper, olive, red pepper, and tomato. |
| Murphy et al. (1991) | The identity of the odorants are not revealed, though they were apparently drawn from a large set (83) and had been rated for familiarity. |
| Doty et al. (1994) | Easy to name microencapsulated odorant that were drawn from the University of Pennsylvania Smell Identification Test (Doty, Shaman and Dann, 1984; Doty, Frye and Agrawal, 1989) were presented to participants (as in Bromley & Doty, 1995) : Pizza, orange, peanut, grape gasoline, cinnamon, licorice, pine, coconut, wintergreen, rose, fruit punch, dill pickle, onion, banana, lime, menthol, chocolate, rose, peach, clove, lime, smoke. |
| Jehl et al. (1994) | The unfamiliar odorants were 36 single aliphatic and aromatic compounds were qualitatively diverse in that they could be described by a large range of olfactory notes (Arctander, 1969): anisic (anisole), balsamic (methyl cinnamate), camphoraceous (3,3,5-trimethyl cyclohexanone), creamy (veratrol), earthy (fenchyl alcohol), ethereal (dipropyl ketone), fruity (iso-amyl acetate, d-limonene), floral (alpha-ionone, beta-phenylethanol), gassy (para-cymene), green (cumene, iso-hexenol), herbaceous (carvacrol, coumarin, thymol), metallic (benzophenone), minty (dextro-camphor, l-menthone), musty (cyclopentanone), rancid (n-butyric acid), rooty (safrole), tarry (naphthalene), and woody (Iinalool). |
| Annett et al. (1995) | Household odors were presented as memory targets or distractors: Acetone, Vinegar, Wintergreen, Savlon, Ammonia, Mint, Coffee, Strawberry, Whiskey, Meths, Aftershave, Amyl acetate, Brasso, Herbs/spices, Honey, Garlic, Cheese, Mustard, Rosewater, Pine, Onion, Beer, Fish, TCP, Petrol, Naphthalene, Orange, Sauce, Aniseed, Turpentine |
| Annett & Lorimer (1995) | Common household odorants comprised memory targets or distractors: Vinegar, Blank, Whiskey, Brasso, Methylated spirit, Tinned salmon, Burnt Toast, Wintergreen, Cigarettes, Chocolate, Mint sauce, Orange, Pine, Strawberry, Peanut, Mixed spices, Baby powder, Coffee, Dog food, Aftershave, Ketchup, Rosewater, Pipe tobacco, Cheese, Lemon, Bleach, Banana, Shoe polish, Onion, Apple, Garlic, Mustard, and Shampoo |
| Bromley & Doty (1995) | The stimuli used isointensive microencapsulated odorants from the University of Pennsylvania Smell Identification Test (Doty, Shaman and Dann, 1984; Doty, Frye and Agrawal, 1989), a smell identification test with micro-encapsulated odorants that are easy to name: Pizza, orange, peanut, grape gasoline, cinnamon, licorice, pine, coconut, wintergreen, rose, fruit punch, dill pickle, onion, banana, lime, menthol, chocolate, rose, peach, clove, lime, smoke. |
| Doty et al. (1995) | For the Odor Recognition Memory Test, 12 microencapsulated “target” odorants were presented, along with 36 foils; although not specifically identified, all the smells were drawn from the University of Pennsylvania Smell Identification Test (Doty, Shaman and Dann, 1984; Doty, Frye and Agrawal, 1989), making them relatively familiar and easy to name. |
| White & Treisman (1997) | The odors selected for the memory experiment were relatively similar in pleasantness, intensity, and familiarity. The smells were synthetic representations of odorants created by flavor and fragrance houses that had a low probability being correctly identified and generated few labels a pilot labelling experiment: Apple, cedarwood, cinnamon, Jamaica rum, lime, mango, methyl salicylate, peach, pineapple, and vanillin. |
| Dade et al. (1998) | Six odors were presented, though the identity of those odorants was not revealed in the paper. |
| White et al. (1998) | The stimuli consisted of 20 familiar odors that had been given names specific to this experiment. Some were supplied by International Flavors and Fragrances: maltballs (chocolate), pie (apple), melon (cantaloupe), porange, (rose), rye (caraway), peat (cedar), sap (pine), and lar (leather). Other odors were produced from commercially available products: flint (Colgin Liquid Smoke), mint (r-Carvone), wintergreen (methyl salicylate), grintergreen (grape juice), mothballs (naphthalene), tar (Humco coal tar solution), fap (cod liver oil), anise (trans-anethol) , lemon (McCormick food flavoring), and orange (McCormick ' food flavoring). Still other odors were produced from the actual materials: beet (Sunnycrest canned beets) and tranis (Wegman's 8 O'clock Coffee). Some of the olfactory foils were not similar enough to each other (mint and wintergreen; peat and sap; rye and anise), so they were mixed to achieve a higher level of similarity. |
| Miles & Jenkins (2000) | The odorants were almond, butter, cherry, chocolate, lemon, orange, raspberry, peppermint, and strawberry, and were specifically named for participants. |
| Reed (2000) | The olfactory stimuli (across experiments) consisted of commercially available perfume oils (Body Shop). The odors were called Heartfelt, White Musk, Oceanus, Vanilla, Strawberry, Ananya, Dewberry, Pot Pourri, and Woodland Spice. All of these scents were judged to be qualitatively different by an independent tester. |
| Dade et al. (2001) | Participants were exposed to peach, geraniol, eucalyptus oil, costus oil, patchouli oil, cinnamon bark oil for the described study, but they also participated in other studies that involved 30 additional (not listed) odorants from similar categories (fruity, floral, minty, unpleasant, woody, spicy) in order to decrease the effectiveness of verbal labels. |
| Danthiir et al. (2001) | The stimuli were the same microencapsulated odors as in Doty et al. (1995), which came from the University of Pennsylvania Smell Identification Test (Doty, Shaman and Dann, 1984; Doty, Frye and Agrawal, 1989) and are thus easily named. |
| Dade et al. (2002) | Four odors from each of 12 categories (Citrus, Fruity, Woody, Balsam oils, Grassy, Minty, Spice, Anise, Light floral, Heavy floral, Animal-like, and Unpleasant) were used, for a total of 48 odors (Grapefruit, Lime oil, Bergamot, Orange oil, Peach, Allyl caproate, Guave, Amyl acetate, Patchouli, Sandlewood oil, Vetyver, Mousse de Chene, Hydrocarboresin, Peru balsam, Labdanum oil, Fir balsam, *Cis*-3-hexanol, Galbanum, Lentisque, Viridine, Eucalyptus oil, Peppermint oil, L-Carvone, Wintergreen, Cinnamon bark oil, Nutmeg oil, Ginger oil, Pepper oil, Anisic aldehyde, Fennel oil, Star anise oil, Tarragon oil, Geraniol, Muguet, Lilial, Freesia, Mimosa, Jonquille, Narcisse, Jasmine, Indole pure, Ambrarome, Castoreum, Civet, Costus oil, 2-Methyl butyric acid, Isovaleric acid, Butyric acid). In order to reduce the effectiveness of verbal labels, the distractor odors used in testing were chosen from the same categories as the memory target odors. |
| Choudhury et al. (2003) | The odorants used in this test—amyl acetate, phenyl ethyl alcohol, peppermint, and peanut— were micro-encapsulated and of similar intensity. They were previously employed in the University of Pennsylvania Smell Identification Test (Doty, Shaman and Dann, 1984; Doty, Frye and Agrawal, 1989) and are thus easily named. |
| Levy et al. (2003) | The stimuli for this study are not fully reported. The stimuli consisted of 28 distinctive odors comprised of common foods, condiments, and household items, but except for three odorants (garlic powder, almond extract, shoe polish), the identity of the stimuli are not disclosed by the authors. |
| Zucco et al. (2003) | The 60 pleasant and odorants in this study are not fully disclosed by the authors. The stimuli were almost all the same as those used by Lyman and McDaniel (1986), and included Almond, Brut after-shave, Clay, Lemon, Pipe tobacco, Soap, Vanilla, and Vicks. The identity of the other 52 odorants was not revealed. |
| Dacremont & Valentin (2004) | For the memory test, 48 odors from everyday life were used: Amber, English sweet, Lemon, Quince, Apple, Eucalyptus, Licorice, Raspberry, Apricot, Fennel, Lily of the valley, Rose, Banana, Fig, Litchi, Soap, Biscuit, Grapefruit, Melon, Tea, Blackcurrant, Green grass, Mint, Tobacco, Black Pepper, Hay, Orange, Truffle, Brioche, Hazelnut, Orange blossom, Sea, Caramel, Honey, Peach, Strawberry, Chocolate, Honeysuckle, Pine, Vanilla, Cinnamon, Lavender, Pineapple, Violet, Coconut, Leather, Prune, Wood fire |
| Miles & Hodder (2005) | Across all seven experiments, the stimuli were: White Musk, Potpourri, Oceanus, Vanilla, Spirit of Moonshine, Blackcurrant, Strawberry, Mixed Spice, Lavender, Lemon, Chocolate, Coffee, Banana, Mixed Herbs, Peppermint, Licorice, and seven not-easily-named odorants (Stable/Horses, Coconut, Washday, Gingerbread, Pineapple, Havana Cigar, and Mahogany). |
| Andrade & Donaldson (2007) | The stimuli were comprised of commercially produced essential aromatherapy oils (bergamot, camomile, lavender, myrrh, eucalyptus, frankincense, tea tree, peppermint, pine, patchouli, rosemary, and lemon) that were diluted in sweet almond base oil. |
| Johnson & Miles (2007) | A set of 120 non-food-related odorants supplied by Dale Air Limited, UK, was employed: Alpine laundry powder, Baby powder, Beauty soap, Bergamot, Boiler room, Bouquet, Brewery, Burning peat, Burnt wood, Camomile, Cannon, Carbolic soap, Caribbean holiday, Cedar wood, Christmas tree, Church incense, Cinnamon, Clinic/hospital, Cloisters, Coal face, Coal fire, Coal gas, Coal/soot, Cut grass, Deep heat, Dentist–clove oil, Dinosaur, Dirty linen, Dragon’s breath, Earthy, Eau de cologne, Egyptian mummy, Eucalyptus, Factory, Farmyard, Fish market, Flatulence, Flowery, Forest, Fox, Freesia, Fresh air, Frosty, Garden shed, Grass/hay, Gun smoke, Havana cigar, Hawaiian, Heather/bracken, Honeysuckle, Hyacinth, Incense, Iron smelting, Jaguar spray, Jasmine, Lavender, Leather, Leather/hide, Lemon eucalyptus & mint, Machine oil, Man-o-war, Methane, Mahogany, Mixed spice, Mountain heather, Mummy, Mustard gas, Musty, Oak, Old drifter, Old inn, Old smithy, Old river, Out at sea, Ozone, Peat, Pencil shavings, Peppermint, Phosgene gas, Pine, Pineapple plantation, Pine/heather/peat, Pit ponies, Polish-wax, Pot-pourri, Riverbank, Rope/tar, Roses, Rotten egg, Rubbish acrid, Sandalwood, Sea breeze, Sea shore, Ships canon, Smoke, Sports rub, Stable/horses, Star’s dressing room, Steam/oil/ships, Steam/oil/trains, Street bomb, Sun sand, & coconut, Swamp, Sweaty feet, Sweet peas, Tobacco leaf, Train smoke, Tropical, Tropical rainforest, Urine, Victoria lavender, Violets, Volcano, Vomit, Wallflower, Washday, Wild stag, Wine cask-oak, Woodsmoke, Ylang jasmine and myrrh. It should be noted that many of the odors were not easily labeled. |
| Doty et al. (2008) | Short-term odor memory/discrimination was assessed using a 12-item four-alternative forced-choice test (Odor Memory Test™, Sensonics, Inc., Haddon Heights, NJ) featuring isointensive microencapsulated odorants from the University of Pennsylvania Smell Identification Test (Doty, Shaman and Dann, 1984; Doty, Frye and Agrawal, 1989). As such, these odorants are typically easily named. |
| Yeshurun et al. (2008) | Thirty odorants that had been rated for nameability and usage were used in the study (manufactured by either Sigma-Aldrich or Sensale): α,α-dimethylphenethyl butyrate, Banana essence, Blue cheese essence, Bornyl acetate, Cheetos snack essence, Cola essence, Ethyl decanoate, Eucalyptus essence, Fresh baguette essence, Fresh cut grass essence, Grapes essence, Heptyl alcohol, Hexyl hexanoate, Hydroxycitronellal, Isoamyl phenylacetate, Iso-phorone, Lemon essence, Licorice essence, Maple syrup essence, Methyl anthranilate, Methyl hexanoate, Methyl octanoate, Mint essence, Nonanoic acid, Nonyl acetate, Peanut butter essence, Pentanol, Rose essence, Watermelon essence, β-ionone (mainly trans) |
| Johnson & Miles (2009) | The same set of 120 odorants used in Johnson and Miles (2007) was employed: Alpine laundry powder, Baby powder, Beauty soap, Bergamot, Boiler room, Bouquet, Brewery, Burning peat, Burnt wood, Camomile, Cannon, Carbolic soap, Caribbean holiday, Cedar wood, Christmas tree, Church incense, Cinnamon, Clinic/hospital, Cloisters, Coal face, Coal fire, Coal gas, Coal/soot, Cut grass, Deep heat, Dentist–clove oil, Dinosaur, Dirty linen, Dragon’s breath, Earthy, Eau de cologne, Egyptian mummy, Eucalyptus, Factory, Farmyard, Fish market, Flatulence, Flowery, Forest, Fox, Freesia, Fresh air, Frosty, Garden shed, Grass/hay, Gun smoke, Havana cigar, Hawaiian, Heather/bracken, Honeysuckle, Hyacinth, Incense, Iron smelting, Jaguar spray, Jasmine, Lavender, Leather, Leather/hide, Lemon eucalyptus & mint, Machine oil, Man-o-war, Methane, Mahogany, Mixed spice, Mountain heather, Mummy, Mustard gas, Musty, Oak, Old drifter, Old inn, Old smithy, Old river, Out at sea, Ozone, Peat, Pencil shavings, Peppermint, Phosgene gas, Pine, Pineapple plantation, Pine/heather/peat, Pit ponies, Polish-wax, Pot-pourri, Riverbank, Rope/tar, Roses, Rotten egg, Rubbish acrid, Sandalwood, Sea breeze, Sea shore, Ships canon, Smoke, Sports rub, Stable/horses, Star’s dressing room, Steam/oil/ships, Steam/oil/trains, Street bomb, Sun sand, & coconut, Swamp, Sweaty feet, Sweet peas, Tobacco leaf, Train smoke, Tropical, Tropical rainforest, Urine, Victoria lavender, Violets, Volcano, Vomit, Wallflower, Washday, Wild stag, Wine cask-oak, Woodsmoke, Ylang jasmine and myrrh |
| Zelano et al (2009) | The supra-threshold odorants in these studies were: amyl acetate, octanoic acid, decyl-alcohol, chocolate oil, benzyl-alcohol, orange oil, L-carvone, benzaldehyde, phenyl-ethyalcohol, garlic, octanol, and mixtures of these compounds. |
| Jönsson et al. (2011) | The odorants in these experiment were comprised of half that were poorly named and half that were easily named: Tridec-2-enenitrile (TDN), 2-Ethenyl-2-methyl benzenepropanal (CIW), 2-Phenylethyl pentyl ether (PPE), Dec-9-en-1-ol (DEO), 2-Phenyl ethyl ethyl ether (PEE), Capronaldehyde, Methylacetate, Althea, 1,4-Methano-7-methyl-(2H)-octahydronaphtalene-6-one (PLI), 3,7-Dimethyloctanenitrile (DON), Orange oil, 7- Methyl (2H,4H)-1,5,benzodioxepin-3-one (CAL), Smoked, Methyl benzoate (MBE), Bornyl acetate (BOR), Butterscotch, Coconut, Almond, Pineapple, Lemon, Lavender, Banana, Peppermint, Anise |
| Valentin et al. (2011) | Both common and uncommon odorants (all provided by Firmenich and diluted in propylene glycol) were used in these experiments: Oxidized, Isocyclemone, Allicisceous, Honey, Boiled, Ripe, Soy beany, Bergamote, Baked, Iodine, Fried, Milky, Citrus, Phenylethyl acetate, Herbal, Trichlorodecenyl isobutyrate, Molten, Damascenone, Almondy, Ozonil, Oily, Paracymene, Fatty savoury, Waxy, Buttery, Lilial, Caramelic, Herbal undecanone, Brothy, Santal, Earthy, Melon valerate, Eggy, Melon heptanal, Blue cheese, Cardboard, Cereal, Bomyl acetate, Anisic, Irone, Tropical, Floral pyranol, Alcoholic, Malty, Yeasty, Menthyl acetate, Cultured, Farenal, Woody, Raw, Berry, Lyral, Rindy, Candied, Fishy, Alcohol C8, Garmay, Gardenial pentyl acetate, Spicy savoury, Skinny Savoury, Minty, Alpha ionone, Jammy, Cardamome, Vanillic, Alpha pinene + camphene, Lemon, Linalool, Eucalyptus, Custard, Peach, Sulfury sweet, Encens, Tagete, Apple, Yiang, Fulfury savoury, Fermented, 8‐Mercapto‐3‐P‐menthanone, Burnt, Cheesy, Ocean propanal, Nutty, Rancid |
| Zucco et al. (2011) | Suprathreshold olfactory stimuli that were comprised of common household odorants, essences, or essential oils were used; all were neat, or dissolved in either distilled water or mineral oil. The stimuli were: Banana, boot grease, almond, juniper, strawberry, Cinnamon, camphor, anchovy, paste, lavender, suntan, cream, Cloves, fish, barley, mustard, tar, Leather, ink, chamomile, nutmeg, tea, Lemon, garlic, coconut, onion, thyme, Liquorice, gasoline, coffee, paint, tomato, Orange, oregano, fennel, pine, vanilla, Peppermint, pineapple, glue, rosemary, vinegar, Rose, shoe cream, honey, rum, violet, Tobacco, turpentine, jasmine, soap, yeast. Wines were also used in this study: Cannonau, Cabernet Sauvignon, Merlot, Refosco, Sangiovese, Arneys, Grey Pinot, Sauvignon,Verdicchio, and White Pinot. |
| Johnson et al. (2013) | The stimuli comprised 10 odor cubes (Dale Air Ltd, UK) selected at random from a corpus of 120 non-food-related odors, presumably the same odorants that were used in Johnson & Miles (2009), though this is not explicitly stated. |
| Lenk et al. (2014) | Various intensities of hydrogen sulphide (H2S, odour of rotten eggs) were used in the study |
| Doty et al. (2015) | The Odor Discrimination/Memory Test [OMT] in this study was a 12-item test developed by Choudhury et al. (2003) and contained four odorants—amyl acetate, phenyl ethyl alcohol, peppermint, and peanut— that were micro-encapsulated and of similar intensity. They were previously employed in the University of Pennsylvania Smell Identification Test (Doty, Shaman and Dann, 1984; Doty, Frye and Agrawal, 1989) and are thus easily named. |
| MacQueen & Drobes (2017) | The 22 household ground spices (High Quality Organics; Reno, NV) that comprised the OST are not explicitly listed, except for Marjoram, Paprika, Fennel, Cumin, Beet, Oregano, and Rosemary. |
| Moss et al. (2018) | The experimental stimuli comprised 160 food and non-food related odorants selected from a corpus of 200 odorants, prepared by Dale Air Ltd. ([www.daleair.com](http://www.daleair.com)); only 20 of those odors (that were previously rated as difficult to verbalize) were explicitly listed: Boiler Room, Man-o'-War, Racing Car, Ginger, Coal Pit, Blue Cheese, Farmyard, Casbah, Coriander, Frosty, Tea Leaf, Basil, Toffee Apple, Clover, Rhubarb, Lychee, Pine/Heather/Peat, Cinnamon, Hot Stuff Male, Black Pepper, Aniseed Balls, Pear, Pear Drops, Fruit Punch, Peach Flesh, Tea Tree Oil, Licorice, Garlic, Mango & Sweet Orange, Sage, Chocolate (Mint), Soap Suds, Fish, Pineapple, Onion, Irish Cream, Rosemary, Coal Soot, Rubber, Train Smoke. |
| Moss et al. (2019) | The odorants were selected from a corpus of 200 food and non-food related odorants, prepared by Dale Air Ltd. (www.daleair.com), on the basis of normative scores reported by Moss et al. (2016); half of the stimuli presented were randomly selected from the 20 highest verbalizability scores and the other half from the 20 lowest verbalizability scores: Carbolic Soap, Sandalwood, Nutmeg, Cuban Cigar Smoke, Nag Champa, Wood Chip, Cinder Toffee, Eucalyptus, Garden Mint, Lime, Marzipan, Pear, Spearmint, Sports Rub. |
| Wenzel et al. (2021) | The roughly iso-intense stimuli included sweet and savory common spices, as well as uncommon odors: Clove, Nutmeg, Ginger, Oregano, Basil, Rosemary, 5-methylheptan-3-one oxime, Vigaflor, Diethyl malonate |
| Yang et al. (2021) | Odors were selected from a library of 10 unique and discriminable odor stimuli (anise oil, cinnamon, coffee, honeysuckle, mint, orange, peanut butter, pine, strawberry, and vanilla). |
| Johnson & Allen (2022) | The odorants for these experiments were selected from a corpus of 200 food and non-food related odorants (AromaPrime Ltd.) based on normative scores reported by Moss et al. (2016): Rum Barrel, Carbolic Soap, Patchouli, Mouse, Sea Shore, Ginger, Burning Peat, Cardamom |
