# Supplementary material for: A cognitive nose? Evaluating working memory benchmarks in the olfactory domain
Source: Chem Senses. 2025 Mar 10;50:bjaf008. doi: 10.1093/chemse/bjaf008 (PMC11985691; doi:10.1093/chemse/bjaf008)
Supplement: bjaf008_suppl_Supplementary_Table_S3 [file bjaf008_suppl_supplementary_table_s3.docx]

| **Suplementary table 3**  *“A” level WM Benchmarks and the studies covering each benchmark.* | | |
| --- | --- | --- |
| **Benchmark Number** | **Benchmark** | **Studies** |
| **1** | Set-Size Effects on Accuracy | Engen et al 1973;  Jones et al., 1978;  Dacremont & Valentin, 2004;  MacQueen & Drobes, 2017;  Valentin et al., 2011. |
| **2** | Set-Size Effects on Retrieval Latency | - |
| **3** | The Effects of Filled Retention Intervals | Engen et al., 1973;  Jones et al.,1975;  Mair et al., 1980;  Miles & Jenkins, 2000;  Choudhury et al., 2003;  Doty et al., 2008;  Wenzel et al., 2021;  Doty et al., 2015;  Murphy et al., 1991;  Bromley & Doty, 1995;  Wenzel et al., 2021. |
| **4** | Primacy and Recency Effects on Accuracy | Annett & Lorimer, 1995;  White & Treisman, 1997;  Miles & Jenkins, 2000;  Reed, 2000;  Miles & Hodder, 2005;  Johnson & Miles, 2007;  Johnson & Miles, 2009;  Johnson et al., 2013;  Moss et al., 2018;  Yang et al., 2021;  Johnson & Allen, 2022. |
| **5** | Confusions of Target Items with Other Items in a Memory Set | White & Treisman, 1997;  Yang et al., 2021 |
| **6** | Locality Constraint on Transpositions | - |
| **7** | Effects Within and Across Domains in a Multiple Memory-Set Effect | Andrade & Donaldson, 2007 |
| **8** | Disruption of Memory by Processing in the Same Domain | Walk & Johns, 1984;  Andrade & Donaldson, 2007;  Zucco, 2003. |
| **9** | Disruption of Memory by Processing in Another Domain | Walk & Johns, 1984;  Murphy et al., 1991;  Annett et al., 1995;  Zucco et al., 2003;  Miles & Hodder, 2005;  Andrade & Donaldson, 2007;  Moss et al., 2019. |
| **10** | Effect of Cognitive Load of the Processing Demand |  |
| **11** | Phonological Similarity | White et al., 1998. |
| **12** | Effects of Distinctiveness and of Grouping: Grouped Lists are Better Recalled | - |
| **13** | Prioritization of Information in WM: Item-Switch Effects |  |
| **14** | Effects of Chunking | - |
| **15** | Hebb Repetition Effect | Johnson et al., 2013. |
| **16** | Positive Manifold | Doty et al, 2015;  MacQueen & Drobes, 2017;  Danthiir et al, 2001. |
| **17** | Correlation Between WM and Attention Indicators | - |
| **18** | Correlation of WM with Fluid Intelligence | Danthiir et al, 2001. |
| **19** | Dissociable Neural Substrates of Different Content Domains | Dade et al. 1998;  Dade et al 2001;  Dade et al 2002;  Zelano et al., 2009;  Lenk et al., 2014. |
| **20** | Preserved WM in Amnesia | Eskenazi et al 1983  Levy et al., 2003. |
| **21** | Measures of Neural Activity Track Amount of Information in WM |  |
